# Phantoms for use in optical coherence elastography

**DOI:** 10.1117/1.JBO.31.8.086004

**Published:** 2026-08-08

**Authors:** Farzan Navaeipour, Jiayue Li, Shima Zamani, Lena Kranold, Rowan W. Sanderson, Brendan F. Kennedy

**Affiliations:** a BRITElab, Harry Perkins Institute of Medical Research, QEII Medical Centre Nedlands and Centre for Medical Research, The University of Western Australia, Perth, Australia; b The University of Western Australia, School of Engineering, Department of Electrical, Electronic and Computer Engineering, Perth, Australia; c The University of Western Australia, School of Engineering, Perth, Australia; d Nicolaus Copernicus University in Toruń, Institute of Physics, Faculty of Physics, Astronomy and Informatics, Torun, Poland

**Keywords:** optical coherence elastography, optical coherence tomography, phantoms, silicone, agar, gelatin, surface roughness, viscoelasticity, optical attenuation.

## Abstract

**Significance:**

Optical coherence elastography (OCE) is an emerging biomedical imaging technique for mapping the micro-scale mechanical properties of tissue. Phantoms are vital for assessing OCE imaging performance in a controlled and systematic manner. Although the use of phantoms in OCE has been widely demonstrated, there is no consensus on suitable phantom materials or fabrication methods, limiting reproducibility and inter-laboratory comparison of OCE techniques.

**Aim:**

Our aim is to establish a unified framework for selecting, fabricating, and characterizing OCE phantoms by systematically evaluating the mechanical, optical, and structural properties of silicone, agar, and gelatin, the three most widely used OCE phantom materials.

**Approach:**

We conducted a literature review of phantom fabrication methods reported in OCE studies published between 1998 and 2025, comprising 223 papers, and identified silicone, agar, and gelatin as the most widely used OCE phantom materials. We experimentally characterized the elasticity, viscoelasticity, and attenuation coefficient of homogeneous phantoms fabricated from each material and investigated the effects of shelf life and optical scatterer concentration on mechanical properties. We further fabricated inclusion phantoms and tissue-mimicking surface roughness phantoms derived from optical coherence tomography (OCT) scans of human breast tissue using all three materials and evaluated their imaging performance using a compression OCE technique, quantitative micro-elastography (QME).

**Results:**

The elastic (tangent) modulus ranged from 7.2 to 175.9 kPa for silicone, 9.7 to 229.1 kPa for agar, and 3.5 to 29.4 kPa for gelatin, while OCT attenuation coefficients ranged from 0.2 to $13.1\;{\mathrm{mm}}^{-1}$ across the three materials. Stress relaxation testing revealed distinct viscoelastic signatures, with relaxation time constants ranging from ∼2  s in silicone to over 1600 s in gelatin. We demonstrated that optical attenuation in these materials can be varied independently of mechanical properties. Inclusion and surface roughness phantoms were successfully fabricated from all three materials, with QME measurements revealing material-specific fabrication artifacts and demonstrating that surface topography can produce erroneous mechanical contrast in mechanically uniform materials.

**Conclusions:**

This study provides a framework to guide phantom material selection, fabrication, and characterization in OCE, identifying silicone as best suited for durable, predominantly elastic phantoms; agar for viscoelastic phantoms across a broad elasticity range; and gelatin for soft, highly viscoelastic phantoms. We believe that the results presented here will support standardization in OCE phantom development and will enable more detailed analysis, validation, and comparison of OCE techniques.

## 1 Introduction

Optical coherence elastography (OCE) is an emerging biomedical imaging technique that combines optical coherence tomography (OCT) with mechanical loading to map the micro-scale mechanical properties of tissues in three dimensions.^1^ OCE has shown promise in diverse applications. For example, in oncology, OCE has shown potential in intraoperative tumor margin assessment.^2^^–^^4^ In ophthalmology, OCE has been developed to quantify corneal biomechanics to guide keratoconus treatment planning and to monitor therapeutic response.^5^^–^^7^ OCE has also demonstrated potential for characterizing atherosclerotic plaques in cardiovascular disease^8^ and for investigating cellular mechanics in mechanobiology.^9^^,^^10^ OCE involves three main steps: applying a mechanical load to the sample, measuring the resulting deformation using OCT, and relating the measured displacement to mechanical properties using mechanical models from continuum mechanics.^11^^,^^12^ OCE techniques are often classified based on the mechanical excitation method used, with compression OCE,^13^^,^^14^ transient OCE,^15^ and harmonic OCE,^16^ being the three most prominent techniques. In the development of OCE, analogously to ultrasound elastography and magnetic resonance elastography,^17^^,^^18^ phantoms are essential for optimization and validation of the measured mechanical properties and to train users to achieve consistent, repeatable measurements.

Phantoms are well-calibrated samples that facilitate characterization of important system parameters including spatial resolution,^19^ sensitivity,^20^ and image contrast.^21^ They have been used in the demonstration of almost all OCE techniques.^22^^–^^25^ However, the rationale for choosing specific phantom designs in OCE studies has not been clearly established, resulting in a wide variety of phantoms being reported in the literature. This lack of standardization makes it challenging to compare different OCE methods and to properly assess imaging performance.

OCE phantoms are characterized by their mechanical, optical, and structural properties.^26^ It is generally desirable to control the mechanical properties in the range of soft tissues.^27^^,^^28^ Such control is often achieved by varying the material concentrations, through multi-component mixing strategies, e.g., by adding non-curing silicone oil to silicone phantoms or the percentage of agar to water in agar phantoms.^29^ For optical properties, normally, the main requirement is to provide optical scattering in the range of biological tissues to ensure adequate OCT signal-to-noise ratio (SNR).^30^^,^^31^ In this paper, we define system characterization phantoms to be phantoms that do not aim to replicate the structural properties of tissues but instead are designed to have well-defined structures that enable OCE system parameters, such as spatial resolution, sensitivity, and contrast-to-noise ratio, to be characterized. Such phantoms include homogeneous cylinders, layered phantoms, and inclusion phantoms. Tissue-mimicking phantoms describe a separate group of phantoms that aim to accurately recreate tissue structure. To date, the majority of OCE phantoms have been system characterization phantoms. However, as OCE techniques mature and as focus shifts toward clinical translation, tissue-mimicking phantoms are likely to become more prominent to analyze and optimize clinical imaging performance. Phantoms have been demonstrated to replicate breast ducts^32^ and tissue surface roughness^33^ and have been used to assess, in a controlled manner, how tissue structure impacts OCE image formation.

Despite phantom fabrication techniques being extensively described in many studies, evaluation of the mechanical, optical, and structural properties across different materials remains limited. For example, mechanical testing performed across OCE studies reports inconsistent protocols, strain rates, and phantom geometries. In addition, optical scatterer concentrations are often reported without characterization of the corresponding OCT attenuation coefficient, making it challenging to compare results obtained from different phantoms and to replicate these results. This variability, combined with the absence of a systematic characterization of structured phantom fabrication with different materials, hinders inter-laboratory reproducibility and prevents the establishment of standardized reference materials for the validation of OCE techniques.

To address these gaps in the characterization of OCE phantoms, we implemented an experimental framework to comprehensively characterize OCE phantoms. Based on the analysis of 223 OCE publications from 1998 to 2025, silicone,^20^^,^^21^^,^^32^^–^^107^ agar,^108^^–^^180^ and gelatin^181^^–^^221^ were selected for experimental characterization. These three materials have been used in 92% of OCE phantoms reported.

First, we characterized the mechanical and optical properties of homogeneous phantoms with a cylindrical geometry. Homogeneous phantoms are the most widely reported in OCE studies reporting new techniques, as they enable the baseline imaging performance to be established prior to demonstration on structured phantoms and tissue. Here, phantoms were fabricated with different material concentrations to allow the elasticity to be varied in a controlled manner. Different optical scattering particles were incorporated at varying concentrations to provide tunable optical properties. These phantoms underwent comprehensive mechanical characterization using uniaxial compression testing,^121^^,^^222^ with tangent moduli ranging from 7.2 to 175.9 kPa for silicone, 9.7 to 229.1 kPa for agar, and 3.5 to 29.4 kPa for gelatin. Mechanical parameters, including elastic moduli, viscoelastic time constants, and viscosity coefficients, were quantified through stress relaxation testing and generalized Maxwell model fitting.^223^^–^^225^ For each material, optical characterization was achieved using OCT-based attenuation coefficient measurements^226^^,^^227^ to establish the relationship between scatterer concentration and optical properties, with OCT attenuation coefficients ranging from 0.2 to $13.1\;{\mathrm{mm}}^{-1}$. Next, we implemented system characterization phantoms. Specifically, we investigated inclusion phantoms and tissue-mimicking phantoms that replicate the surface roughness of freshly excised human breast specimens.^33^ To achieve this, we used a previously reported compression OCE technique, quantitative micro-elastography (QME).^84^ Using this approach, we aim to provide guidelines for phantom material selection and structural design for use across OCE techniques.

## 2 Literature Review

To establish guidelines for phantom fabrication in OCE, a literature review was conducted. This analysis provides a foundation for material selection and the establishment of fabrication protocols that address phantom requirements across OCE studies.

**Fig. 1:**
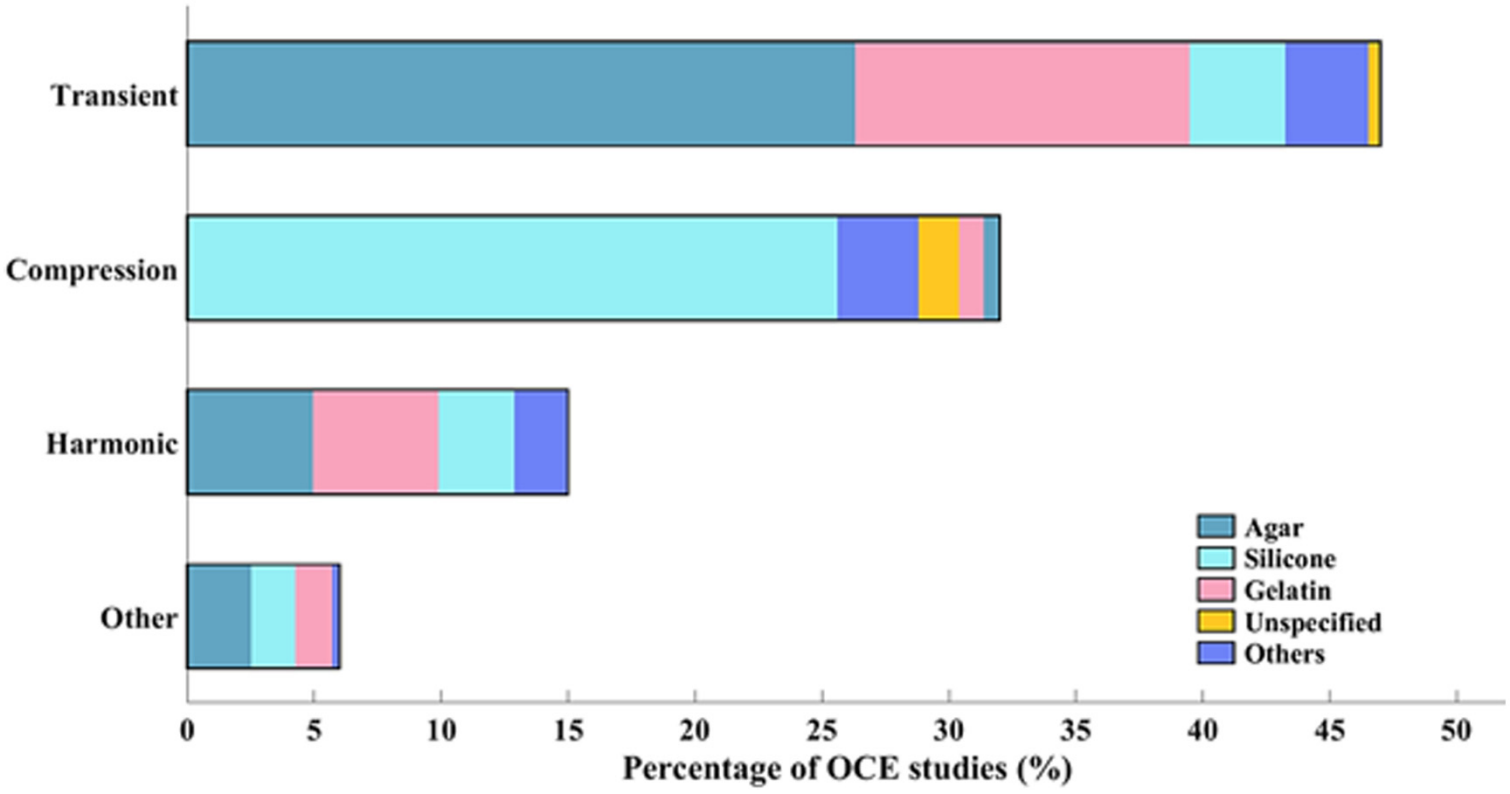
Bar charts showing the phantom materials used in the most prominent OCE techniques. These data were derived from 223 papers published between 1998 and 2025. The bars represent the different OCE techniques used, grouped as transient OCE, compression OCE, harmonic OCE, and other OCE techniques, with the length of each bar indicating the prevalence of each technique. The color-coded segments within the bars indicate the material used: agar (dark teal), silicone (light teal), gelatin (pink), unspecified materials (yellow), and others (purple).

A search was conducted in the Web of Science database covering the period 1998 to 2025. The search was restricted to title, abstract, and keyword fields using the terms “optical coherence elastography,” “quantitative micro-elastography,” “OCT and elastography,” and “optical coherence tomography and elastography.” Only original research articles were considered, with review papers, book chapters, and conference proceedings excluded. This process identified 700 publications, from which studies not involving phantoms, or where the phantoms used were not adequately described, were removed, resulting in 223 papers. The OCE techniques, phantom materials, structural designs, optical scattering agents, and mechanical property ranges used in these papers were recorded and are summarized in Fig. 1 and Table 1, with the complete dataset provided in Table S1 in the Supplementary Material.

### 2.1 Phantom Materials Used in OCE Techniques

The phantom materials used for the three most prominent OCE techniques are presented in Fig. 1. In OCE studies involving phantoms, the most widely used technique was transient OCE (47%), followed by compression OCE (32%) and harmonic OCE (15%). The three most commonly used phantom materials were silicone (used in 35% of studies), agar (used in 36% of studies), and gelatin (used in 21% of studies). Together, these materials accounted for 92% of phantoms used in OCE. The remaining 8% comprised materials including polyvinyl alcohol,^19^^,^^228^^–^^235^ polyacrylamide,^236^^–^^238^ silica gel,^239^^–^^243^ and other materials^244^^–^^256^ (grouped as “others” indicated with purple color in Fig. 1).

Agar was the most frequently employed phantom material in transient OCE: used in 56% of studies (59 out of 105 papers). Silicone was used in 80% of compression OCE studies (58 out of 72 papers). Gelatin showed broader distribution across OCE techniques, appearing in many transient OCE studies (28%, 29 out of 105 papers) and harmonic OCE studies (33%, 11 out of 34 papers).

### 2.2 Phantom Structural Designs

Table 1 summarizes the main phantom parameters identified from previous OCE papers, with these parameters listed for each of the three main phantom materials. Silicone phantoms demonstrated the greatest structural versatility, implemented in homogeneous (41%), inclusion (39%), and layered (17%) system characterization phantoms, as well as small numbers of tissue-mimicking designs, including vessel,^42^ duct,^32^ and breast cancer^32^^,^^33^ phantoms. This versatility stems from silicone’s moldability, mechanical stability, and compatibility with multi-component casting processes. Agar has only been used for system characterization phantoms, with homogeneous (60%), layered (30%), and inclusion (10%) designs. Similarly, gelatin has only been used to fabricate system characterization phantoms, comprising homogeneous (56%), layered (37%), and a limited number of inclusion designs (7%).

#### 2.2.1 Optical scattering agents

Correct selection of an optical scattering agent and its concentration is important in OCE, as it determines image quality and imaging depth. Interestingly, despite its importance, 93 of the 223 papers (42%) did not specify the optical scattering of the phantoms used, highlighting the need for more standardization and description of OCE phantom fabrication methods.

When reported, the selection of the scattering agent demonstrated material-dependent preferences. In silicone phantoms, titanium dioxide (${\mathrm{TiO}}_{2}$) was the most widely used scattering agent (68%), with concentrations ranging from 0.02% to 1.3% w/v, where both particle size and refractive index are the key determinants of the optical scattering achieved. This concentration range provides OCT attenuation coefficients in the range of soft biological tissues, which have been reported to be between 1.6 and $16.5\;{\mathrm{mm}}^{-1}$.^31^ In 26% of silicone phantoms, no scattering agents were added, with the intrinsic scattering from the silicone being sufficient. Less common use of other scatterers was also reported, including polystyrene beads and alumina powder (collectively 6%). Throughout this paper, concentrations of solid scattering agents are reported as weight per volume (w/v) and those of liquid scattering agents as volume per volume (v/v).

In 50% of agar phantoms, no scattering agent was reported. When scatterers were added, Intralipid was the most common (23%), with concentrations ranging from 0.25% to 20% v/v. The prevalence of Intralipid in agar phantoms is attributed to its water solubility, biocompatibility, and ease of integration with hydrogel matrices. Milk was also employed (11% of agar phantoms) at concentrations of 0.5% to 3% v/v. ${\mathrm{TiO}}_{2}$ usage in agar phantoms was limited (10%) with concentrations ranging from 2% to 20% w/v, due to the higher density of ${\mathrm{TiO}}_{2}$ causing the particles to aggregate at the bottom of the solution during the gelation process. Other scattering agents, including graphite powder, ink, and latex microspheres, accounted for a combined 6% of agar phantom studies.

Gelatin phantoms displayed the most diverse scattering agent distribution. In 42% of gelatin phantoms, no scatterers were added. Of the remaining gelatin phantoms, ${\mathrm{TiO}}_{2}$ was used in 20% of cases in concentrations of 0.05% to 0.2% w/v, followed by Intralipid in 16% of cases in concentrations of 0.25% to 10% v/v, and silicon carbide in 9% of cases in concentrations of 0.05% to 0.3% w/v. Other scattering agents (black paint, milk, latex microspheres, and charcoal particles) were employed in 13% of gelatin phantoms. Material-specific preferences reflect underlying compatibility requirements. ${\mathrm{TiO}}_{2}$ integrates well with silicone matrices, Intralipid is compatible with hydrogel systems, and gelatin is suitable for use with a range of scattering agents.

**Table 1:** Summary of OCE phantom characteristics for the three most widely used materials.

Phantom material	OCE technique	Phantom structure	Optical scattering agent	Scatterer concentration used	Young’s modulus range (kPa)
Silicone (78 studies)	Compression (73%) Transient (10%) Harmonic (9%) Other^b^ (8%)	Homogeneous (41%) Structured (59%)	${\mathrm{TiO}}_{2}$ (68%)	0.02% to 1.3% w/v	3 to 5000
			No scatterer added (26%)	—	
			Others (6%)		
Agar^a^ (80 studies)	Transient (70%) Others (14%) Harmonic (13%) Compression (3%)	Homogeneous (60%) Structured (40%)	No scatterer (50%) Intralipid (23%) Milk (11%) ${\mathrm{TiO}}_{2}$ (10%) Others (6%)	— 0.25% to 20% v/v 0.5% to 3% v/v 0.02% to 0.2% w/v	5 to 600
Gelatin (47 studies)	Transient (63%) Harmonic (21%) Others (14%) Compression (2%)	Homogeneous (56%) Structured (44%)	No scatterer added (42%) Intralipid (16%) ${\mathrm{TiO}}_{2}$ (20%) Silicon carbide (9%) Others (13%)	— 0.25% to 10% v/v 0.05% to 0.2% w/v 0.05% to 0.3% w/v	3 to 120

a Agarose is included in this category.

b Studies employing multiple OCE techniques simultaneously are included in the “others” category.

### 2.3 Mechanical Property Ranges

The last column in Table 1 summarizes the range of Young’s modulus, which is defined as bulk axial stress divided by bulk axial strain, reported for silicone, agar, and gelatin phantoms. Young’s modulus of soft tissues has been reported to be in the range from 0.1 to 1000 kPa.^28^ The most common approaches for measuring Young’s modulus are uniaxial compression testing^129^^,^^222^ and tensile testing.^257^^,^^258^ Other less frequently reported techniques include rheometry^259^^,^^260^ and indentation.^261^^,^^262^ Silicone phantoms demonstrated the broadest dynamic range, with Young’s modulus reported from 3 to 5000 kPa. Agar phantoms exhibited Young’s modulus ranging from 5 to 600 kPa. Gelatin phantoms exhibited a range of Young’s modulus between 3 and 120 kPa. Although elasticity has been extensively characterized by measuring Young’s modulus, characterization of the viscoelastic properties across all phantom materials remains limited. This should be addressed to maximize the potential to translate OCE techniques to routine use in biomedical applications, as tissues generally exhibit strong viscoelastic behavior. Although viscoelastic parameters have been measured using each of the three main OCE techniques,^85^^,^^100^^,^^263^ independent characterization of the viscoelastic properties of phantoms, using, e.g., rheology or creep deformation testing, has rarely been reported.

### 2.4 Need for Standardization in OCE

Despite the importance and widespread use of phantoms in OCE studies, key aspects of their design and characterization remain unclear. For example, important parameters, such as mechanical properties and optical attenuation coefficients, are often not specified, making it difficult to compare OCE techniques across different studies. This lack of standardization hinders the translation of OCE to biomedical applications, as it obscures the imaging performance of OCE and the ability to assess the suitability of OCE techniques for use in each application area.

In this study, we addressed the need for standardization in OCE phantoms by providing fabrication protocols and comprehensive characterization of the three main OCE phantom materials across all three phantom types, namely, silicone, agar, and gelatin. We believe that this study will provide a straightforward means to better determine the most suitable OCE phantom. First, homogeneous cylindrical phantoms were characterized to determine elasticity, viscoelasticity, and optical attenuation (Sec. 4.1). Subsequently, heterogeneous system characterization phantoms, in the form of inclusion phantoms, and tissue-mimicking surface roughness phantoms were fabricated and characterized using a compression OCE technique, QME, to assess the mechanical contrast and structural fidelity. Establishing fabrication protocols and characterization data across all three phantom types and all three materials provides a unified reference framework for OCE phantom development. Although surface-roughness-mimicking phantoms have been previously demonstrated in silicone,^33^ to our knowledge, this is the first study to report fabrication protocols and QME characterization of such phantoms using agar and gelatin.

## 3 Materials and Methods

This section details the fabrication protocols and characterization methods used for silicone, agar, and gelatin phantoms. Material-specific preparation procedures are described first, followed by fabrication protocols for system characterization phantoms (homogeneous and inclusion phantoms) and tissue-mimicking surface roughness phantoms. Mechanical and optical characterization protocols are then described. A summary of all phantom compositions used in this study is provided in Table 2.

### 3.1 Phantom Fabrication

#### 3.1.1 Silicone phantom preparation

Silicone phantoms were fabricated using two-part silicone elastomers (Ecoflex 00-20 and Ecoflex 00-45, Smooth-On, Macungie, Pennsylvania, United States), where part A is the silicone base and part B is the catalyst, along with silicone thinner oil (Smooth-On). These components were combined in varying mixing ratios to achieve different mechanical properties. Each phantom batch was prepared by first mixing the silicone base with silicone thinner oil for 5 min to ensure even distribution. A catalyst was then added and mixed for a further 5 min. The mixture was subsequently placed in a vacuum chamber for 5 min to remove air bubbles. To achieve a flat phantom surface, the cylindrical mold was filled so the meniscus of the silicone was slightly above the top edge of the mold. A glass slide was then placed on top, allowing excess mixture to flow over the edge. The phantoms were cured at room temperature (20 to 25°C) for at least 4 h, which was the standard curing condition across silicone phantoms in this study.

To provide optical scattering in silicone phantoms, ${\mathrm{TiO}}_{2}$ (catalog no. 232033, anatase powder, average particle size ∼0.6  μm, 99.8% trace metals basis; Sigma-Aldrich, St. Louis, Missouri, United States) was added at varying concentrations, as described in Table 2. The ${\mathrm{TiO}}_{2}$ preparation protocol involved first placing the particles in an oven at 70°C for 1 min to remove residual humidity, which is unwanted as it promotes agglomeration. The dried ${\mathrm{TiO}}_{2}$ powder was then added to the silicone base and dispersed using an ultrasonic bath for 10 min to break up agglomerates. A minimum stirring time of 1 h is required to achieve adequate dispersion of scatterers, with higher ${\mathrm{TiO}}_{2}$ concentrations requiring extended mixing times up to 6 h. In this study, all mixtures were stirred for more than 6 h prior to adding catalyst to ensure adequate dispersion of scatterers for all concentrations. The catalyst was then added and mixed for 5 min before pouring the mixtures into molds.

#### 3.1.2 Agar phantom preparation

Agar powder (A7921, Sigma-Aldrich) was weighed at the desired concentration and was then dispersed in deionized water. The mixture was stirred continuously at room temperature for 5 min to ensure even distribution of agar granules and was then gradually heated to 90°C to 95°C on a magnetic stirrer hot plate until complete dissolution was achieved. The solution was maintained at 70°C for 1 h to prevent premature gelation and to allow air bubbles to dissipate. The mixture was then de-gassed under vacuum for ∼3  min before being poured into molds. The mold surfaces were coated with a thin layer of acrylic spray (White Knight, Clayton, Australia) as a release agent to facilitate phantom removal.

To introduce optical scattering, Intralipid (20% stock emulsion, Sigma-Aldrich) was used as the scattering agent. The desired Intralipid concentration was first prepared by diluting the appropriate volume of stock solution in deionized water to achieve the final concentration (for example, a 40% v/v Intralipid concentration consisted of 40% Intralipid solution and 60% deionized water by volume). The mixture was stirred thoroughly to ensure uniform Intralipid dispersion prior to adding the agar powder, after which the fabrication steps described above were followed.

#### 3.1.3 Gelatin phantom preparation

Gelatin powder (type A, 175 g Bloom; Sigma-Aldrich) was gradually mixed into deionized water under gentle stirring to ensure even hydration, and the mixture was allowed to bloom at room temperature for 30 min to enable full hydration of the gelatin granules. Then, the solution was heated to 60°C on a magnetic stirrer hot plate until complete dissolution was achieved (∼10 to 15 min). The solution was subsequently de-gassed under vacuum for 3 min to remove air bubbles before being poured into molds.

${\mathrm{TiO}}_{2}$ and Intralipid were both used to introduce optical scattering to gelatin phantoms. ${\mathrm{TiO}}_{2}$ powder was added to deionized water and stirred for 2 min to achieve a well-dispersed suspension of scatterers. Gelatin powder was then added gradually and allowed to bloom. The mixture was then cast following the same procedure as described above. For gelatin phantoms containing Intralipid, the desired concentration was prepared by diluting the appropriate volume of stock solution in deionized water and stirred for 1 min before adding gelatin powder. The mixture was stirred prior to the blooming step. After this, the same fabrication procedure as described above was followed.

**Table 2:** Material concentrations and optical scatterer specifications for the different phantom types. For each formulation, three phantoms were fabricated. For silicone and gelatin, three repeated measurements were performed per phantom (nine measurements per formulation); for agar, a single measurement was performed per phantom (three measurements per formulation).

Phantom type	Material	Mixing ratio and concentrations	Scattering agent	Scatterer concentrations
Homogeneous phantoms	Silicone (Ecoflex 00 to 20) Silicone (Ecoflex 00 to 45)	1:1:2 1:1:1 1:1 2:1 1:1	${\mathrm{TiO}}_{2}$	0.05% (w/v) 0.1% (w/v) 0.2% (w/v) 0.4% (w/v) 0.6% (w/v) 0.8% (w/v) 1.0% (w/v) 1.5% (w/v) 2.0% (w/v)
	Agar	0.5% (w/v) 1% (w/v) 1.5% (w/v) 2% (w/v) 3% (w/v)	Intralipid	0.5% (v/v) 1% (v/v) 2% (v/v) 6% (v/v) 10% (v/v) 15% (v/v) 20% (v/v) 30% (v/v) 40% (v/v)
	Gelatin	7% (w/v) 10% (w/v) 15% (w/v) 20% (w/v) 25% (w/v)	${\mathrm{TiO}}_{2}$	Same as above ${\mathrm{TiO}}_{2}$ concentrations
			Intralipid	Same as above Intralipid concentrations
Inclusion phantoms	Silicone bulk (Ecoflex 00-20)	1:1:1	${\mathrm{TiO}}_{2}$	0.05% (w/v)
	Silicone inclusion (Ecoflex 00-20)	2:1	${\mathrm{TiO}}_{2}$	0.4% (w/v)
	Agar bulk	1% (w/v)	Intralipid	1% (v/v)
	Agar inclusion	2% (w/v)	Intralipid	6% (v/v)
	Gelatin bulk	10% (w/v)	${\mathrm{TiO}}_{2}$	0.05% (w/v)
	Gelatin inclusion	20% (w/v)	${\mathrm{TiO}}_{2}$	0.4% (w/v)
Surface roughness phantoms	Silicone (Ecoflex 00 to 20)	1:1:1	${\mathrm{TiO}}_{2}$	0.2% (w/v)
	Agar	1% (w/v)	Intralipid	4% (v/v)
	Gelatin	20% (w/v)	${\mathrm{TiO}}_{2}$	0.2% (w/v)

### 3.2 System Characterization Phantoms

#### 3.2.1 Homogeneous phantoms

To create homogeneous phantoms, following the material preparation procedures described in Secs. 3.1.1 to 3.1.3 for each material, the solution was poured into cylindrical molds with an aspect ratio of 1:1, i.e., a diameter of 10 mm and a height of 10 mm. This aspect ratio was chosen to minimize boundary effects during compression testing.^264^ Silicone phantoms were cured at room temperature for at least 4 h. Agar phantoms were allowed to cool at room temperature for 2 to 3 min then refrigerated at 4°C for at least 2 h to ensure complete gelation. Gelatin phantoms were left to gel at room temperature for 30 min then stored at 4°C for at least 4 h to ensure full solidification.

For all three materials, the softest formulations were limited by the minimum concentration needed for the material to solidify. The stiffest silicone formulation was selected to provide sufficient coverage of the elasticity range of soft tissues relevant to OCE applications, rather than because of fabrication limitations. In contrast, the stiffest agar and gelatin formulations were constrained by rapid solidification at higher concentrations, which caused the solutions to solidify before they could be adequately degassed and poured into the molds.

After demolding, silicone phantoms were stored at room temperature, while agar and gelatin phantoms were stored in sealed containers at 4°C to prevent dehydration prior to characterization. Three phantoms per material type and concentration were fabricated to enable statistical analysis. Although agar phantoms were tested immediately upon transfer from 4°C to room temperature, gelatin phantoms were equilibrated at room temperature for 1 h prior to mechanical testing to ensure consistent measurement conditions.

#### 3.2.2 Inclusion phantoms

Inclusion phantoms were fabricated for all three materials using the preparation procedures described in Secs. 3.1.1 to 3.1.3. All details of material and optical scatterer concentrations for inclusion phantoms are provided in Table 2. Each phantom was fabricated following a three-step casting procedure described in detail previously for silicone inclusion phantoms^83^^,^^98^ to produce a phantom with a stiffer, more highly scattering disk-shaped inclusion fully embedded within a softer homogeneous bulk matrix. The inclusion was designed to be stiffer than the bulk to provide mechanical contrast detectable by QME and more highly scattering to provide optical contrast allowing the inclusion to be resolved in OCT intensity images.

Most inclusion phantoms demonstrated so far for use in OCE have been created using silicone, with only several examples of agar or gelatin inclusion phantoms.^34^^,^^151^^,^^156^^,^^183^^,^^192^ Given that inclusion phantoms are needed to characterize OCE system parameters such as resolution and sensitivity, the aim of our study is to establish the feasibility of making reliable inclusion phantoms using agar and gelatin, benchmarked against silicone inclusion phantoms.

To fabricate the phantoms, a thin base layer of the bulk matrix material was first cast in a cylindrical mold (diameter: 6 mm; thickness: ∼0.3  mm) and cured. The cylindrical inclusion (diameter: 1 mm; thickness: 0.5 mm) was cut from a separate cast and cured layer of inclusion material using a circular punch. The inclusion was placed on top of the cured base layer at the center of the cylinder, and additional bulk matrix material was poured over the inclusion to fill the mold completely before curing to its final thickness, producing a phantom with the inclusion embedded ∼300  μm below the surface of a bulk cylinder.

### 3.3 Surface Roughness Phantoms

To demonstrate tissue-mimicking phantoms, we fabricated surface roughness phantoms that replicated the topography of biological tissue, specifically excised human breast tissue.^33^

Silicone and agar phantoms were fabricated following the preparation procedures described in Secs. 3.1.1 and 3.1.2, with the addition that a specialized cylindrical mold was used, where the bottom surface replicated the surface profile of a human breast tissue specimen. The molds were three-dimensional (3D)-printed following the methodology described by Sanderson et al.,^33^ whereby the surface topography of breast tissue was segmented from volumetric OCT scans of the tissue. Molds were printed on a form 2 stereolithography printer (Formlabs, Somerville, Massachusetts, United States) using Clear Resin V4 at a layer height of 25  μm.

For surface roughness phantoms fabricated using gelatin, a two-step molding approach was employed. Demolding was difficult to achieve for gelatin phantoms without damaging the phantom, even with the use of mold release agents, including acrylic spray and silicone oil, likely because of interfacial adhesion between gelatin and the surface of the 3D-printed plastic mold.^265^ Our solution was to first 3D print a negative of the original plastic mold and cast silicone in this mold. The cured silicone was then used as the mold for gelatin fabrication, as gelatin does not adhere to silicone surfaces, enabling reproducible demolding without damaging the phantoms.

### 3.4 Mechanical Characterization Using Uniaxial Compression Testing

#### 3.4.1 Uniaxial compression testing

Mechanical characterization was achieved using a custom-made uniaxial compression testing system, controlled via LabVIEW (Version 21, National Instruments, Austin, Texas, United States).^38^ Phantoms were positioned between two rigid plates. The lower plate was attached to a load cell (LSB200, FUTEK, Irvine, California, United States) and a motorized axial translation stage (MLJ050/M, Thorlabs Inc., Newton, New Jersey, United States), while the upper plate was rigidly fixed in position. Prior to testing, both the upper and lower contact surfaces of phantoms were lubricated with silicone oil (AK50, Wacker, Munich, Germany) to minimize friction during compression.

Cylindrical phantoms were placed on the lower plate and brought into initial contact using the translation stage. From this position, the phantom was compressed by moving the translation stage at a strain rate of 0.5%/s. The applied force was measured continuously using the load cell, with a safety cutoff set at 15 N to avoid overloading. Axial strain was calculated from the displacement of the translation stage relative to the initial phantom height (engineering strain), while axial stress was determined by dividing the measured force by the initial phantom cross-sectional area (engineering stress).

#### 3.4.2 Elasticity characterization through stress–strain testing

Following uniaxial compression testing, the acquired stress–strain data were processed using a custom MATLAB script (R2023b, MathWorks, Natick, Massachusetts, United States) to estimate the tangent modulus of the phantoms. The tangent modulus, defined as the slope of the stress–strain curve at a given strain, was used as the measure of phantom elasticity and is reported at 10% strain throughout this study. Under the assumption of linear elasticity, which is generally reasonable at 10% strain, tangent modulus is equivalent to Young’s modulus.^266^ Three phantoms of each formulation (i.e., each mixing ratio for silicone, and each agar and gelatin concentration, as detailed in Table 2) were tested. For silicone and gelatin, three repeated measurements were performed per phantom (total nine tests per formulation). For agar, phantoms were tested only once (a total of three tests per formulation), as they did not revert to the initial height after loading. This was attributed to irreversible deformation or rearrangement of the gel network along with fluid exudation from the agar matrix under compression,^267^^,^^268^ consistent with the poroelastic behavior previously reported for agar.^269^ Agar phantoms were compressed to a maximum strain of 20% due to plastic deformation at higher strain levels, while silicone and gelatin phantoms were compressed to 50% strain.

For each compression test, raw stress–strain curves were imported into the custom-built MATLAB script for processing. To model the nonlinear stress–strain response while avoiding artifacts that arise when fitting exponentials to data that do not pass exactly through the origin, each individual stress–strain curve was fit using a double-exponential model with a constant offset^270^

$$y=ae^{\left(bx\right)}+ce^{\left(dx\right)}+f_{0},$$ (1)

where y represents the stress, x represents the strain, a and c represent the magnitudes of the two exponential stress components, and b and d determine how rapidly each component increases with strain. The term $f_{0}$ is a constant offset. During fitting, a, b, c, and d were constrained to be non-negative, while $f_{0}$ was left unconstrained. This prevents the fit from producing non-physical tangent modulus values at higher strains. The tangent modulus, representing the instantaneous slope of the stress–strain curve at any given strain level, was calculated as the derivative of the fitted exponential model

$$dy/dx=abe^{\left(bx\right)}+cde^{\left(dx\right)}.$$ (2)

Notably, the constant offset term $f_{0}$ does not affect the tangent modulus. This approach yields a continuous description of strain-dependent stiffness. To enable statistical comparisons, all fitted stress and tangent modulus curves were interpolated onto a common strain grid, so that measurements from different tests could be compared at the same strain values. At each strain value, the mean and standard deviation (SD) of tangent modulus were calculated across all tests, yielding continuous mean ± SD curves as a function of strain. The tangent modulus curves were used as reference measurements for comparison with OCE measurements, described in Secs. 4.2.1 and 4.2.2, respectively.

#### 3.4.3 Viscoelasticity characterization through stress relaxation testing

Stress relaxation testing was performed using the uniaxial compression testing system described in Sec. 3.4.1 to characterize the viscoelastic properties of silicone, agar, and gelatin phantoms under controlled conditions. Prior to each test, the interface between the phantoms and the rigid compression plates was lubricated with 10-μL silicone oil to minimize friction.^44^ Each phantom was compressed to 20% strain, the maximum strain tolerated by agar before fracture. This strain also ensured a sufficiently large initial stress for reliable relaxation measurements across all materials and formulations. Following compression, the strain was maintained at this value, while the stress was recorded. The hold time was 2 min for silicone and agar and 30 min for gelatin, as gelatin required longer to reach equilibrium due to its higher viscoelasticity.^271^^,^^272^

Three phantoms were prepared for each material, with three tests performed on each of the silicone and gelatin phantoms, yielding nine measurements for each of these phantoms. Tests were conducted in a rotating sequence: the first test was performed on all three phantoms sequentially, followed by the second repeat on all phantoms, and then the third repeat. This protocol ensured adequate recovery time (5 to 6 min) among consecutive tests on the same phantom. Consistent with stress–strain testing protocols, gelatin phantoms were equilibrated at room temperature for 1 h before testing. Agar phantoms were tested only once due to the material degradation described in Sec. 4.1.1.

Viscoelastic behavior is often described using rheological models composed of elastic springs and viscous dashpots, arranged either in series or parallel.^273^^,^^274^ For viscoelastic solids, stress relaxation is often modeled using the generalized Maxwell model (also known as the Wiechert model), which consists of a number of Maxwell elements (a spring and dashpot in series) placed in parallel with a single spring.^223^^,^^224^ The generalized Maxwell model represents the material response as the sum of individual Maxwell elements, each with distinct relaxation times that contribute to the overall stress relaxation behavior. Maxwell models comprising one or two Maxwell elements can adequately quantify the viscoelastic behavior of materials such as silicone, agar, and gelatin while remaining computationally manageable.^225^

The stress–time data were fitted to a generalized Maxwell model with two Maxwell elements in parallel with an isolated spring to characterize the time-dependent mechanical behavior of each phantom material. Model selection was validated by comparing single-element and two-element configurations. The single-element model yielded goodness of fit ($R^{2}$) values between 0.94 and 0.95, while the two-element model achieved $R^{2}>0.99$ for all phantom materials and formulations tested. The mathematical representation of the model used in this study is

$$\sigma (t)=\sigma_{e}+\sigma_{1}e^{\left(-t/\tau_{1}\right)}+\sigma_{2}e^{\left(-t/\tau_{2}\right)}.$$ (3)

Dividing Eq. (3) by the initial applied strain, $\varepsilon_{0}$, gives the relaxation modulus

$$E(t)=E_{e}+E_{1}e^{\left(-t/\tau_{1}\right)}+E_{2}e^{\left(-t/\tau_{2}\right)},$$ (4)

where σ(t) is the time-dependent stress, $\sigma_{0}$ is the initial stress, $\sigma_{e}$ represents the equilibrium stress from the isolated spring (corresponding to the long-term elastic response), $\sigma_{1}$ and $\sigma_{2}$ are the stress contributions from the two Maxwell elements at t=0, and $\tau_{1}$ and $\tau_{2}$ are the corresponding relaxation time constants. The relaxation time constants are related to the material properties through $\tau_{i}=\eta_{i}/E_{i}$, where $\eta_{i}$ is the viscosity coefficient of the dashpot and $E_{i}$ is the elastic modulus of the spring for each Maxwell element.^275^^,^^276^

The equilibrium modulus, $E_{e}=\sigma_{e}/\varepsilon_{0}$, describes the elastic mechanism of the degenerated element with the spring having infinite relaxation time, representing the limiting value of the elastic modulus at t→∞. The instantaneous modulus, $E_{0}=\sigma_{0}/\varepsilon_{0}$, represents the value of the relaxation modulus at t→0, defined as

$$E_{0}=E_{e}+E_{1}+E_{2}.$$ (5)

This generalized Maxwell model captures both the instantaneous elastic response and the time-dependent viscoelastic response of phantom materials. The parameters extracted from the fitting process provide quantitative measures of the elastic moduli, viscous components, and relaxation time constants for each material, enabling comprehensive characterization of their viscoelastic properties under physiological loading conditions.^277^ The instantaneous and equilibrium moduli extracted from stress relaxation testing are distinct from the tangent modulus reported from stress–strain testing. Tangent modulus characterizes the strain-dependent stiffness of a material, while $E_{0}$ and $E_{e}$ quantify the elastic response of the material at the beginning and end of the stress relaxation, respectively. The ratio $E_{e}/E_{0}$ reflects the degree of viscous dissipation during relaxation.

### 3.5 Optical Characterization of Phantoms Using OCT Attenuation Imaging

To characterize the optical properties of phantoms, OCT was used to measure the attenuation coefficient ($\mu_{t}$), which quantifies the rate at which OCT intensity decreases with depth due to scattering and absorption in the phantom.^226^ Importantly, measuring the attenuation coefficient provides a means to not only quantitively control the optical properties of phantoms but also allows phantoms to be fabricated with optical properties close to those of target tissues.

#### 3.5.1 OCT system

All phantom scans were acquired using a spectral-domain OCT system (Telesto III, Thorlabs Inc.). The system utilizes a superluminescent diode light source with a central wavelength of 1300 nm and a bandwidth of 170 nm, providing a measured full width at half-maximum axial resolution of 5.5  μm and a lateral resolution of 13  μm in air. The objective lens had a numerical aperture of 0.04 (LSM04, Thorlabs Inc.). The imaging depth range was ∼3.5  mm in air. The system was configured in common-path mode, where the interface between the imaging window and the phantom surface acts as the reference reflector.^20^ The camera exposure time was set to 15  μs with an A-scan line period of 20  μs, enabling rapid data acquisition while maintaining sufficient SNR.

OCT volumes were acquired over a lateral (xy) area of $8\times 0.5\;{\mathrm{mm}}^{2}$, comprising 800 A-scans per B-scan and 50 B-scans per volume, yielding an effective lateral pixel size of 10  μm in both x and y directions. Phantoms were positioned with their surface in contact with the imaging window to ensure a consistent imaging protocol for all phantoms and to facilitate reproducible measurement of the attenuation coefficient.

#### 3.5.2 Attenuation coefficient measurements

The attenuation coefficient of each phantom was measured from OCT scans following the methodology described by Foo et al.^226^ Briefly, the OCT intensity I(x,y,z) can be modeled using the Beer–Lambert law as

$$I(x,y,z)=\alpha (x,y)S(z)F(x,y,z)e^{\left(-2\mu_{t}\left(x,y,z\right)z\right)},$$ (6)

where α(x,y) is the scaling parameter, S(z) is the spectrometer sensitivity roll-off function, F(x,y,z) is the confocal function, and $\mu_{t}(x,y,z)$ is the attenuation coefficient. The factor of 2 accounts for the double-pass of light in the phantom.

Prior to performing phantom measurements, a calibration scan was acquired using a sample consisting of polystyrene microspheres (0.50  μm diameter, Polybead 07307-15, Polysciences, Inc., Warrington, Pennsylvania, United States) diluted in distilled water (3% v/v), with a relatively low known attenuation coefficient of $0.3\;{\mathrm{mm}}^{-1}$, which was calculated from the Mie theory.^226^ This calibration phantom was used to characterize the sensitivity roll-off function S(z) and confocal function F(x,y,z) of the OCT system. The attenuation coefficient was calculated by correcting the measured OCT intensity for system characteristics and performing a linear fit to the corrected log-transformed intensity. To reduce noise, each A-scan was averaged with ∼10×10 adjacent A-scans in the lateral (x,y) plane, corresponding to an area of $100\;\mu m^{2}\times 100\;\mu m^{2}$. The attenuation coefficient was then extracted from the slope of the fit.

For the optical scatterer concentrations mentioned in Table 2, three identical phantoms were fabricated (described in Sec. 3.1.1) and scanned. For each phantom, 50 B-scans acquired within the volume were averaged to reduce noise, and the attenuation coefficient was then calculated over a $0.15\;{\mathrm{mm}}^{2}(\text{depth})\times 2\;{\mathrm{mm}}^{2}$ (lateral) region of interest within the averaged B-scan. Further details of the attenuation calculation algorithm can be found in Foo et al.^226^

### 3.6 Mechanical Characterization of Structured Phantoms Using QME

QME was performed to characterize the mechanical properties of the fabricated inclusion and surface roughness phantoms. The measurements were conducted using the OCT system described in Sec. 3.5.1. Micro-scale compression was applied at a frequency of 12 Hz using a ring actuator (Piezomechanik GmbH, Munich, Germany) attached to an imaging window. Prior to scan acquisition, each phantom was positioned between a fixed compression plate and the imaging window. A pre-characterized compliant layer was used to estimate stress at the surface of the phantom. The layer was placed on top of the phantom with matched elasticity. The compliant layer had a diameter of 6 mm and a thickness of 500  μm.

To minimize friction at all interfaces during QME scanning, lubricants were applied among the imaging window, compliant layer, phantom, and compression plate.^44^ Silicone oil (AK50) was used for silicone phantoms, while hyaluronic acid (HA) gel (5.8  mg/mL) was used for both agar and gelatin phantoms. A bulk pre-strain was applied to the phantom assembly using a motorized translation stage to ensure even contact among all layers. For inclusion phantoms, a pre-strain of 2% to 3% was applied, while for surface roughness phantoms, a pre-strain of 20% was applied to ensure uniform contact between the layer and the phantom. Local displacement within the phantom was calculated using phase-sensitive detection,^234^ and axial strain was determined as the spatial gradient of displacement with depth using weighted least squares linear regression over a fitting range of 100  μm.^47^ The axial stress at the phantom surface was estimated by combining the measured axial strain at each lateral position of the layer-sample interface with the pre-characterized stress–strain curve of the compliant layer. Assuming uniform and uniaxial stress distribution, the local elasticity was calculated by dividing the local stress in the layer by the local strain in the phantom.^38^

QME volume acquisitions consisted of 500 A-scans per B-scan and 500 B-scan pairs per volume over a lateral field of view of $4\times 4\;{\mathrm{mm}}^{2}$ for inclusion phantoms and 800 A-scans per B-scan and 800 B-scans per volume over a lateral field of view of $12\;{\mathrm{mm}}^{2}\times 12\;{\mathrm{mm}}^{2}$ for surface roughness phantoms. The camera exposure time was set to 20  μs with an A-scan rate of 30  μs, resulting in a total acquisition time of ∼50  s per volume.

To measure the height profile of the surface roughness phantoms, ThorImage software (Thorlabs Inc.) was used. Each phantom was imaged in a dual-arm configuration over a $12\;{\mathrm{mm}}^{2}\times 12\;{\mathrm{mm}}^{2}$ field of view. The acquired OCT volumes were processed using MATLAB to extract surface profiles by performing edge detection at the air-phantom interface. A Canny-based edge detection algorithm^278^ was applied to each B-scan to detect the phantom surface.

## 4 Results

### 4.1 Characterization of Homogeneous Phantoms

#### 4.1.1 Temporal stability of materials

**Fig. 2:**
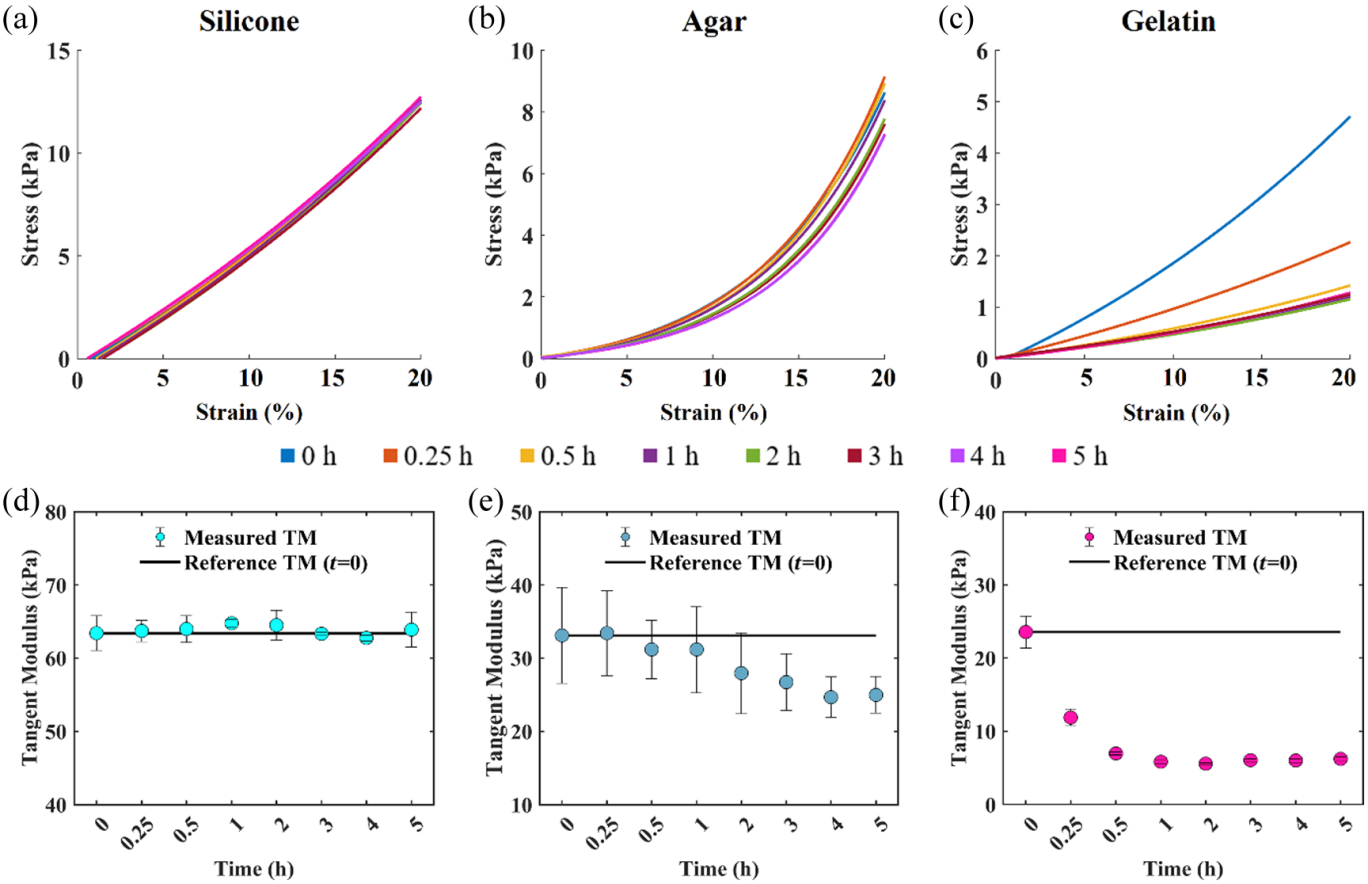
Temporal stability and repeatability of phantom materials at room temperature. (a)–(c) Stress–strain curves and (d)–(f) tangent modulus measured at 10% strain for silicone, agar, and gelatin phantoms, respectively, over a 5-h period. Reference tangent modulus (black line) in panels (d)–(f) indicates the initial measurement at t=0. Error bars represent ± SD of three consecutive compression tests on a single phantom.

Prior to systematic mechanical characterization, we investigated the temporal stability and repeatability of each phantom material at room temperature. This preliminary analysis was essential for establishing appropriate testing protocols that ensure reliable and reproducible measurements across different material types. Figures 2(a)–2(c) present the stress–strain curves, and Figs. 2(d)–2(f) present the tangent modulus measurements at 10% strain for silicone, agar, and gelatin phantoms, respectively, acquired over a 5-h period. Each measurement represents the average of three consecutive compression tests performed on a single phantom.

Silicone phantoms demonstrated consistent stress–strain behavior and temporal stability throughout the 5-h observation period [Fig. 2(a)], with minimal variation in the measured tangent modulus (63.4±2.4  kPa), corresponding to a percentage error of 3.8%, and no noticeable change over time [Fig. 2(d)]. This stability indicates that silicone phantoms are not sensitive to exposure to room temperature and are suitable for repeated testing without an equilibration time.

Agar phantoms exhibit substantial temporal instability and low intra-sample repeatability. Sequential compression tests on the same phantom produced changes in the stress–strain response, with subsequent tests showing reduced stress [Fig. 2(b)]. This behavior is attributed to possible moisture loss or structural damage under compression. In addition, agar phantoms demonstrated temperature sensitivity, with tangent modulus decreasing from 33.1±6.5  kPa at t=0 to 25.0±2.5  kPa after 5 h at room temperature [Fig. 2(e)], representing a 25% reduction in stiffness.

When gelatin phantoms were tested immediately after transferring from refrigeration (4°C), consecutive compression tests on the same phantom yielded decreasing stress after the initial test [Figs. 2(c) and 2(f)]. After 1 h at room temperature, gelatin phantoms achieved stable mechanical properties. The stress–strain curves became closely grouped [Fig. 2(c)] and repeat measurements on the same phantom were consistent, indicating that a room temperature settling period is required to reach equilibrium before testing. The tangent modulus at equilibrium was 5.8±0.2  kPa and remained relatively constant over the remaining period [Fig. 2(f)], with a percentage error of 3.6%.

Based on these findings, material-specific testing protocols were established for all subsequent mechanical characterization experiments. Silicone phantoms were characterized using three independent phantoms with three repeated compression tests per phantom. Agar phantoms were tested using three independent phantoms with a single compression test per phantom to avoid confounding effects of moisture loss and repeated loading. Gelatin phantoms were equilibrated at room temperature for 1 h prior to testing then characterized using three independent phantoms with three repeated compression tests per phantom.

#### 4.1.2 Mechanical characterization

##### 4.1.2.1 Elasticity characterization through stress–strain testing

**Fig. 3:**
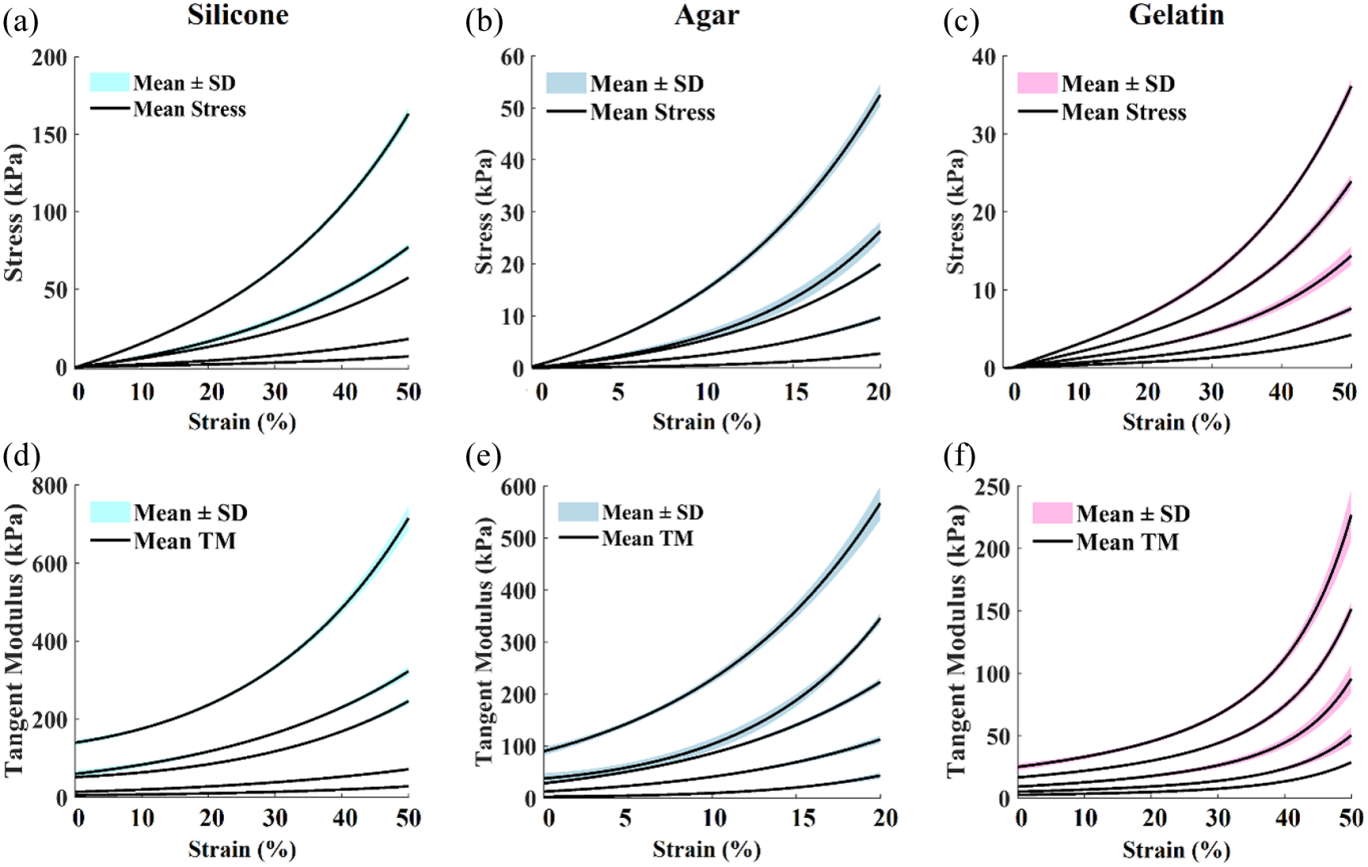
Elasticity characterization of phantom materials via uniaxial compression testing. (a)–(c) Stress–strain curves and (d)–(f) corresponding tangent modulus–strain curves for silicone, agar, and gelatin phantoms, respectively. The top panels show stress as a function of strain, with individual curves representing independent phantoms (silicone: 3 phantoms × 3 repeated tests; agar: 3 phantoms × 1 test; gelatin: 3 phantoms × 3 repeated tests). The bottom panels display strain-dependent tangent modulus curves calculated as the instantaneous slope of the stress–strain curve. Bold lines represent the mean values across all measurements, while shaded regions indicate SD.

Following the establishment of material-specific testing protocols, we performed systematic mechanical characterization of all three phantom materials using uniaxial compression testing. Figure 3 presents the stress–strain relationships and the derived tangent modulus-strain curves for silicone, agar, and gelatin phantoms.

Silicone exhibited the largest range of measured tangent moduli. Each silicone mixture exhibited nonlinear stress–strain and tangent modulus-strain responses over 50% applied strain [Figs. 3(a) and 3(d)]. The corresponding tangent modulus at 10% strain ranged from 7.2±0.4 to 175.9±4.3  kPa (Table 2), representing a 24-fold stiffness variation among the different tested silicones.

Agar phantoms were tested to 20% strain due to plastic deformation at higher strains, consistent with the relatively brittle nature of agar gels.^279^ Within the achievable strain range, agar showed clear concentration-dependent strain-stiffening, with stress rising nonlinearly across formulations [Fig. 3(b)] and tangent modulus also increasing with strain [Fig. 3(e)]. The stress–strain curves were approximately linear up to ∼10% strain, followed by greater strain-stiffening at higher deformations. The tangent modulus at 10% strain increased linearly with agar concentration ($R^{2}=0.97$), ranging from 9.7±0.7  kPa at 0.5% (w/v) to 229.1±7.5  kPa at 3% (w/v) [Fig. 3(e), Table 2]. This relationship between concentration and stiffness aligns well with previous findings, where Young’s modulus of agar phantoms (1.7 to 6.6% w/w) ranged from ∼50 to 450 kPa.^260^

Gelatin phantoms demonstrated nonlinear stress–strain behavior up to 50% strain [Fig. 3(c)], with increasing tangent modulus as strain increased [Fig. 3(f)]. Compared with silicone, gelatin showed a smaller stiffness range across the tested concentrations, with tangent modulus values at 10% strain ranging from 3.5±0.2  kPa (7%, lowest concentration) to 29.4±0.6  kPa (25%, highest concentration). Similarly to agar, we noticed a strong linear relationship between the gelatin concentration and the observed tangent modulus at 10% ($R^{2}=0.98$). The lower gelatin stiffness measured in this study compared with previously reported values is likely due to the testing protocol, as gelatin phantoms were equilibrated at room temperature for 1 h before mechanical testing. As shown in Fig. 2(f), this equilibration caused the phantoms to soften before reaching a stable mechanical state, whereas many previous OCE studies did not report equilibration period prior to testing.

##### 4.1.2.2 Viscoelasticity characterization

**Fig. 4:**
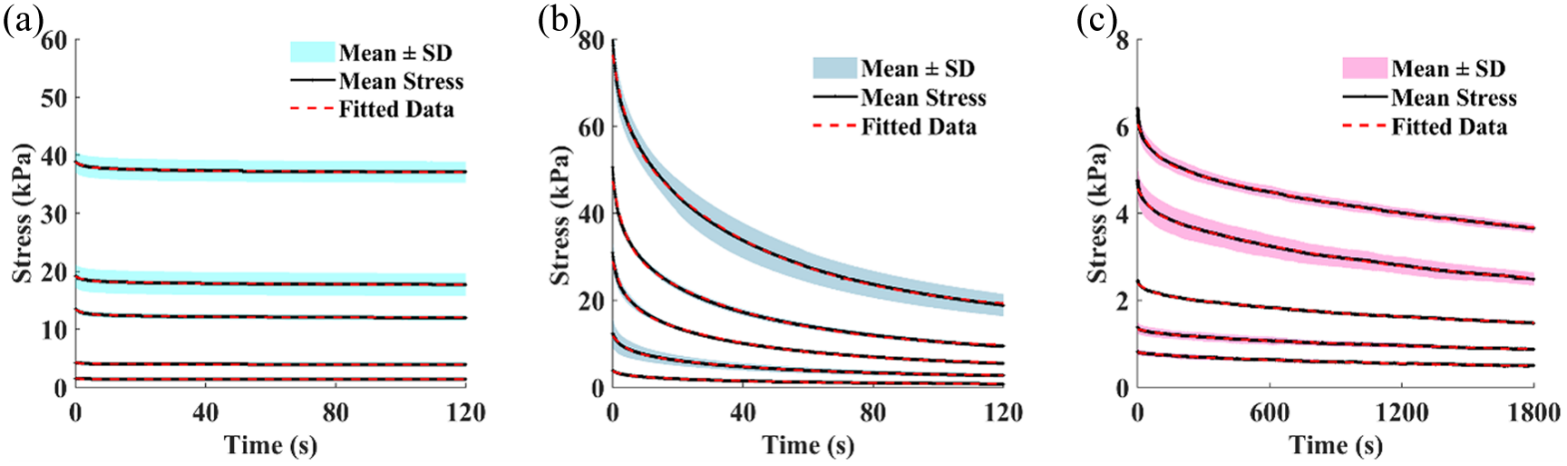
Stress relaxation behavior of phantom materials following rapid compression to 20% strain. Stress as a function of time for (a) silicone, (b) agar, and (c) gelatin phantoms at the formulations listed in Table 2. Within each panel, curves progress from the softest (bottom) to the stiffest (top) formulation. Solid black lines represent the mean values, and shaded regions represent ± SD. Dashed red lines show fits to the two-element generalized Maxwell model ($R^{2}>0.99$ for all fits).

To characterize the time-dependent mechanical behavior of phantom materials, stress relaxation tests were performed following rapid compression (within ∼2  s) to 20% strain. Figure 4 presents the stress relaxation curves for all tested formulations (listed in Table 2), and the parameters extracted from the two-element generalized Maxwell model are provided in Table 3. All materials exhibited stress relaxation, but the extent and timescale of the relaxation differed among silicone, agar, and gelatin, indicating distinct viscoelastic behavior across the three materials. Model selection was validated by comparing single-element and two-element configurations.

Silicone showed the most elastic-dominant response. For all silicone formulations, stress dropped slightly during the first few seconds and then reached an early plateau, indicating that most of the deformation was elastic, rather than dissipated over time. This behavior is also reflected in the close agreement between the initial and equilibrium modulus values, with the equilibrium modulus remaining at ∼90% to 96% of the initial modulus after relaxation. Although stiffer silicone formulations had higher elastic modulus, the relaxation time constants remained within a relatively narrow range ($\tau_{1}=2.3$ to 2.9 s and $\tau_{2}=30.9$ to 36.7 s) indicating that changing formulation primarily altered stiffness rather than the time-dependent response. Overall, these results show that silicone behaves predominantly as an elastic solid with only a small viscoelastic component.

Agar exhibited a substantially greater viscoelastic contribution than silicone. In all agar formulations, stress continued to decrease throughout the 2-min hold period, and the difference between the initial and equilibrium moduli was large, with the equilibrium modulus corresponding to ∼17% to 22% of the initial modulus. This indicates that a considerable proportion of the applied stress was dissipated during relaxation. Agar therefore exhibited substantially more viscous dissipation than silicone.

Gelatin showed the strongest time-dependent behavior of all three materials. Stress decreased progressively over the entire 30-min observation window. Compared with silicone and agar, gelatin exhibited much longer relaxation time constants, with $\tau_{1}∼20$ to 55 times and ∼13 to 35 times greater than silicone and agar, respectively. $\tau_{2}$ was ∼32 to 53 times and ∼25 to 41 times greater than silicone and agar, respectively. The equilibrium modulus remained well below the initial modulus for all formulations, with values at equilibrium corresponding to 46% to 53% of the initial modulus. This indicates that gelatin maintained less stress at equilibrium than silicone but more than agar over the measurement period. Interestingly, lower-concentration gelatin formulations showed longer relaxation times ($\tau_{1}=129.5\;s$ at 7% w/v versus 69.7 s at 25% w/v), suggesting that softer gels relax more slowly.^280^^,^^281^

**Table 3:** Elasticity and viscoelastic parameters of silicone, agar, and gelatin phantoms extracted from mechanical characterization. Tangent modulus (TM) measured at 10% strain from uniaxial compression stress–strain testing, reported as mean ± SD. Initial modulus ($E_{0}$) represents the instantaneous elastic response at t=0, while equilibrium modulus ($E_{e}$) represents the long-term elastic response at t=∞, both extracted from generalized Maxwell model fitting to stress relaxation data acquired at 20% strain. Fast ($\tau_{1}$) and slow ($\tau_{2}$) relaxation time constants. Viscosity coefficients ($\eta_{1}$ and $\eta_{2}$) quantify the energy dissipation associated with each Maxwell element, calculated from $\eta_{i}=\tau_{i}\times E_{i}$. Goodness of fit ($R^{2}$) exceeds 0.99 for all formulations. Values are reported as mean ± SD across nine measurements per formulation for silicone and gelatin and three measurements per formulation for agar.

Phantom	Tangent modulus (kPa)	Initial modulus (kPa)	Equilibrium modulus (kPa)	Time constant (s)		Viscosity coefficient (kPa.s)	
	TM	$E_{0}$	$E_{e}$	$\tau_{1}$	$\tau_{2}$	$\eta_{1}$	$\eta_{2}$
Silicone 00 to 20, 1:1:2	7.2±0.4	7.6	7.0	2.3	30.9	0.9	3.9
Silicone 00 to 20, 1:1:1	19.7±1.1	21.3	19.7	2.8	35.6	2.7	23.1
Silicone 00 to 20, 1:1:0	63.8±1.6	67.1	60.1	2.5	32.4	10.5	90.7
Silicone 00 to 20, 2:1:0	83.7±6.1	95.6	88.5	2.8	36.4	11.0	113.5
Silicone 00 to 45,1:1:0	175.9±4.3	194.0	185.4	3.0	36.7	13.4	147.2
Agar 0.5% (w/v)	9.7±0.7	18.6	3.4	4.6	46.4	25.8	444.7
Agar 1% (w/v)	41.1±2.2	59.4	12.1	3.9	42.3	68.0	1274.3
Agar 1.5% (w/v)	86.3±2.5	144.6	25.1	3.8	39.2	181.1	2814.5
Agar 2% (w/v)	103.8±11.5	236.9	42.3	3.7	40.6	279.5	4860.5
Agar 3% (w/v)	229.1±7.5	382.2	82.4	4.0	43.9	333.1	9513.0
Gelatin 7% (w/v)	3.5±0.2	4.0	1.8	129.5	1625.4	36.7	3063.1
Gelatin 10% (w/v)	6.3±0.1	6.7	3.5	111.5	1619.4	53.2	4362.0
Gelatin 15% (w/v)	12.8±0.8	11.9	6.2	66.2	1338.7	71.6	6116.2
Gelatin 20% (w/v)	23.8±0.5	22.9	10.5	58.3	1167.1	156.9	11,308.6
Gelatin 25% (w/v)	29.4±0.6	30.2	15.3	69.7	1424.1	278.1	15,626.7

#### 4.1.3 Mechanical stability and storage conditions of fabricated phantoms

**Fig. 5:**
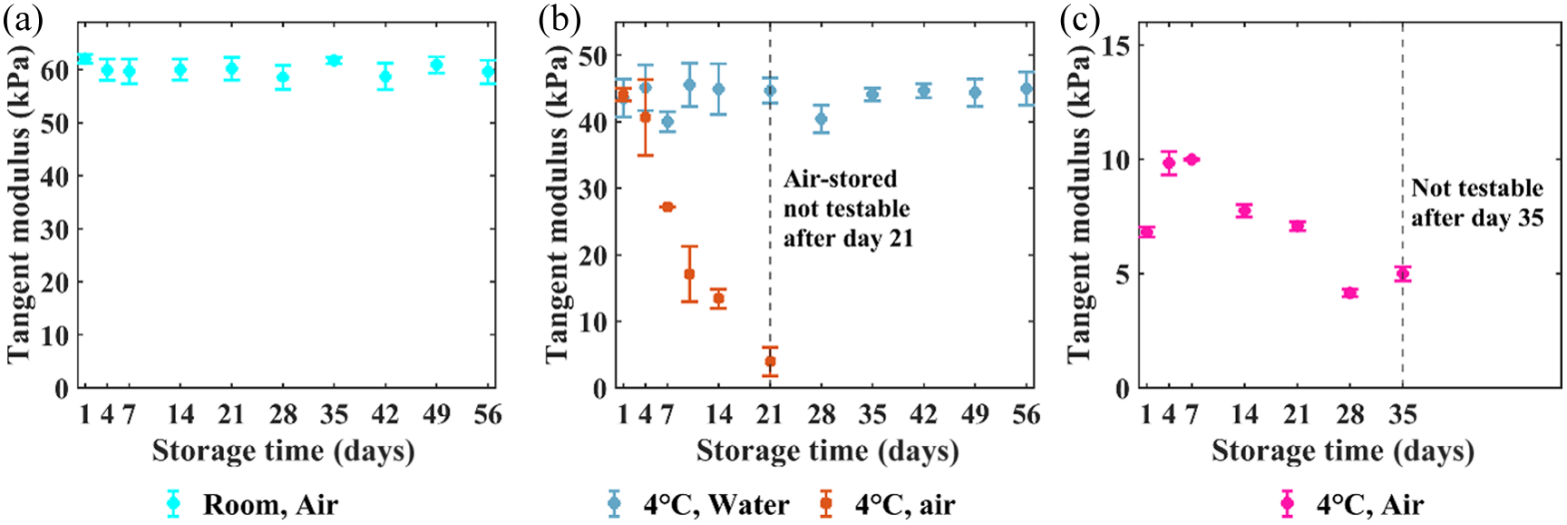
Mechanical stability of phantom materials under different storage conditions. Tangent modulus measured at 10% strain as a function of storage time for (a) silicone phantoms stored at room temperature (20°C to 25°C) in sealed containers, (b) agar phantoms stored at 4°C in sealed containers (green circles) and immersed in deionized water (orange squares), and (c) gelatin phantoms stored at 4°C in sealed containers. At each time point, data points represent the mean ± SD of three measurements: three repeated tests on a single phantom for silicone and gelatin and a single test on each of three phantoms for agar.

To evaluate the long-term mechanical stability of fabricated phantoms under different storage conditions, stress–strain tests were performed on days 1, 4, 7, 14, 21, 28, 35, 42, 49, and 56 for silicone and agar phantoms and at days 1, 4, 7, 14, 21, 28, and 35 for gelatin phantoms. Figure 5 presents the tangent modulus measured at 10% strain as a function of storage time for silicone, agar, and gelatin phantoms under their respective storage conditions.

Silicone phantoms stored at room temperature in sealed containers demonstrated excellent long-term mechanical stability. The average tangent modulus over an 8-week observation period was measured 62.0±0.9  kPa, with error <6% of the initial value. This stability confirms that silicone phantoms can be stored at room temperature without refrigeration, making them highly practical for repeat use.

Agar phantoms were evaluated under two storage conditions: in containers in air at 4°C and immersed in deionized water at 4°C. Phantoms stored in air showed decreasing tangent modulus from 44.1±0.9  kPa at day 1 to 4.0±2.1  kPa at day 21 (90% error relative to day 1), beyond which phantoms became too soft and lost their structural integrity. This change is attributed to syneresis, where water molecules are expelled from the gel network over time, causing shrinking and softening of the phantom.^282^^,^^283^ In contrast, phantoms stored under immersion in deionized water exhibited stable mechanical properties throughout the 56-day observation period, with tangent modulus values ranging from 40.0±1.5 to 45.5±3.2  kPa (∼9% error). Storage under immersion in water prevented dehydration-driven syneresis, which was observed in air.^282^ The results are consistent with a previous study,^260^ which reported that agar-based phantoms stored in distilled water maintain stable mechanical properties for more than 3 months.

Gelatin phantoms were stored in containers at 4°C without water, as water immersion causes gelatin to absorb water and swell, altering both phantom geometry and mechanical properties.^284^^,^^285^ Despite refrigerated dry storage, gelatin phantoms showed considerable variation in tangent modulus over the 35-day observation period, with values ranging from 4.2±0.2 to 7.6±0.2  kPa, beyond which the phantoms disintegrated. These results suggest that gelatin phantoms have limited temporal stability even under refrigerated conditions and should ideally be characterized within 1 to 2 days of fabrication.^286^^,^^287^

#### 4.1.4 Optical characterization of phantoms

**Fig. 6:**
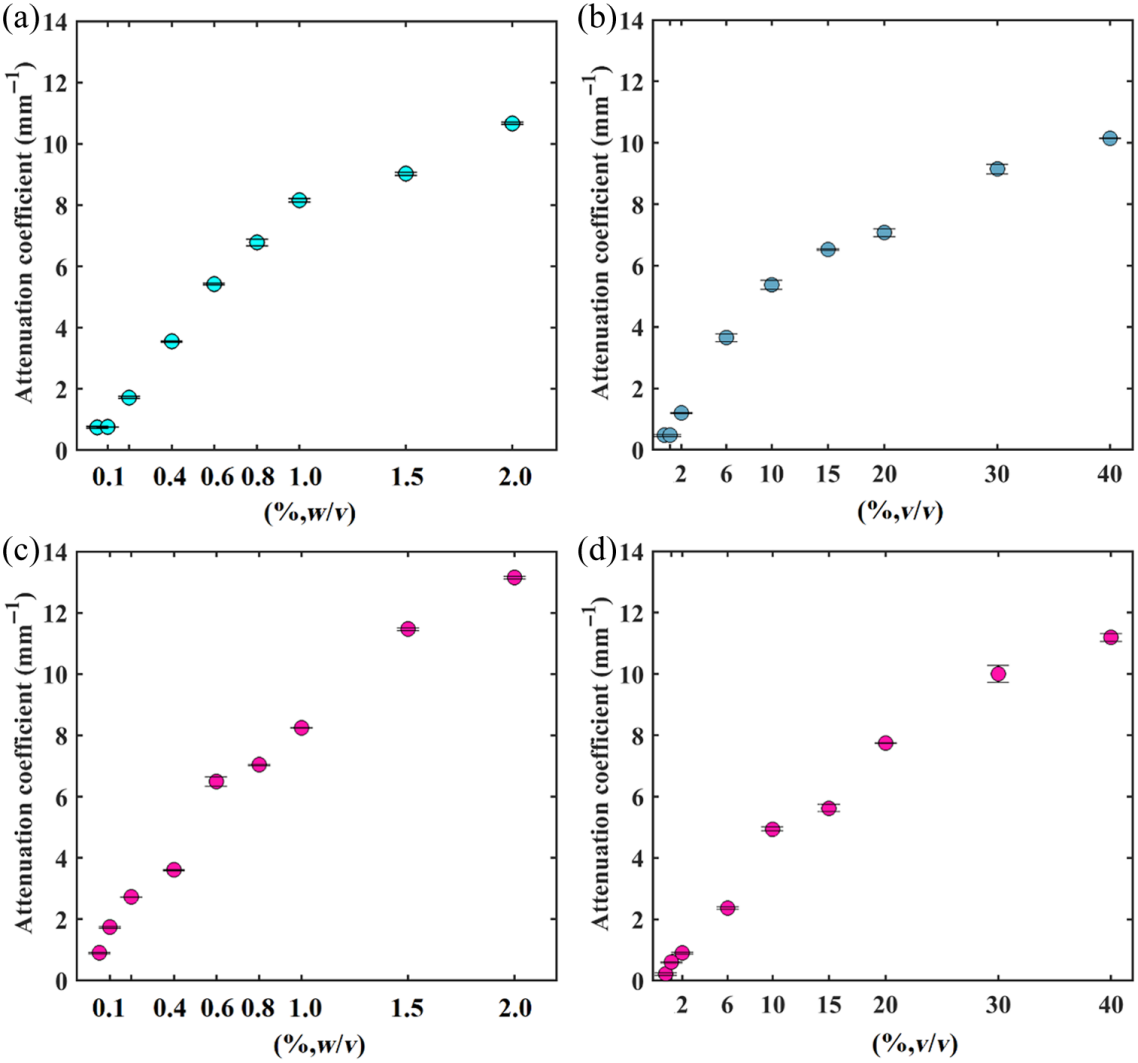
Concentration-dependent optical attenuation in homogeneous phantoms. Attenuation coefficient as a function of scatterer concentration for (a) ${\mathrm{TiO}}_{2}$ in silicone, (b) Intralipid in agar, (c) ${\mathrm{TiO}}_{2}$ in gelatin, and (d) Intralipid in gelatin. Data points represent the mean attenuation coefficient ± SD from three replicate phantoms per concentration.

To characterize the optical properties of phantoms, attenuation coefficients were measured using OCT attenuation imaging as described in Sec. 3.5.2. For each scatterer concentration, three identical phantoms were fabricated and scanned. A region of interest (ROI) was selected in each attenuation map to calculate the mean and SD of the attenuation coefficient in each phantom. Figure 6 presents the measured attenuation coefficients for all three phantom materials with varying optical scatterer concentrations.

The results in Fig. 6 show that the OCT attenuation coefficient increases with scatterer concentration (listed in Table 2), which is consistent across all phantom materials and scatterers used. Silicone phantoms with ${\mathrm{TiO}}_{2}$ [Fig. 6(a)] showed attenuation coefficients increasing from 0.7±0.1 to $10.7\pm 0.1\;{\mathrm{mm}}^{-1}$. Intralipid was not used in silicone due to its incompatibility with hydrophobic elastomeric matrices. Agar phantoms with Intralipid [Fig. 6(b)] exhibited attenuation coefficients ranging from 0.5±0.1 to $10.1\pm 0.1\;{\mathrm{mm}}^{-1}$. ${\mathrm{TiO}}_{2}$ was not characterized in agar due to uneven dispersion in aqueous matrices. Gelatin was compatible with both solid and liquid scattering agents, and similar behaviors were observed. ${\mathrm{TiO}}_{2}$ [Fig. 6(c)] yielded attenuation coefficients from 0.9±0.1 to $13.1\pm 0.1\;{\mathrm{mm}}^{-1}$, while Intralipid [Fig. 6(d)] produced attenuation coefficients from 0.2±0.1 to $11.2\pm 0.1\;{\mathrm{mm}}^{-1}$. The approximately linear relationships between scatterer concentration and attenuation coefficient observed across all material–scatterer combinations enable predictable optical property control. The achievable range of attenuation coefficients (0.2 to $13.1\;{\mathrm{mm}}^{-1}$) encompasses the optical properties of most soft biological tissues.^31^

#### 4.1.5 Effect of optical scattering on tangent modulus

**Fig. 7:**
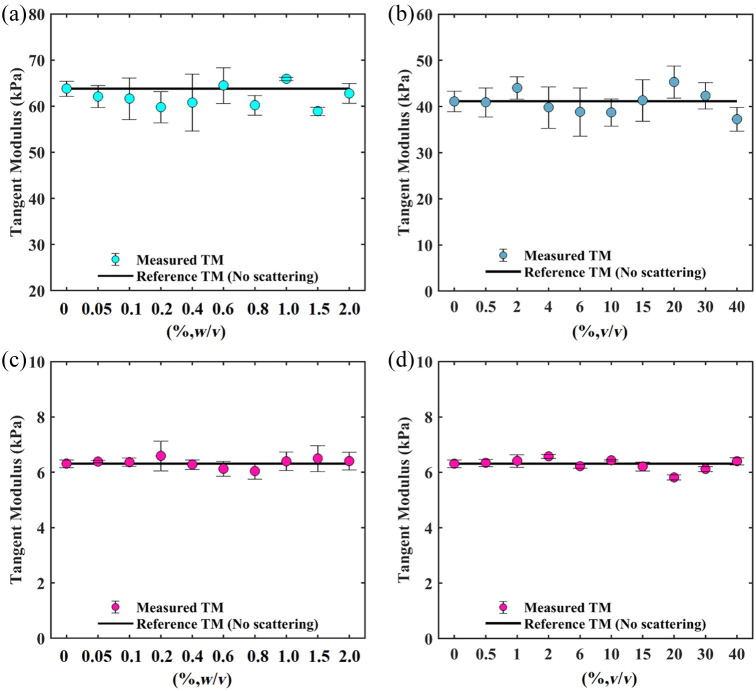
Mechanical stability of phantoms with varying optical scatterer concentrations. Tangent modulus at 10% strain for (a) ${\mathrm{TiO}}_{2}$ in silicone, (b) Intralipid in agar, (c) ${\mathrm{TiO}}_{2}$ in gelatin, and (d) Intralipid in gelatin. Black horizontal lines show the reference values from phantoms without scatterers. Data points represent mean ± SD.

We also investigated whether the addition of optical scatterers affects the measured tangent modulus of phantoms. Figure 7 presents the tangent modulus measured at 10% strain for phantoms containing varying concentrations of optical scatterers, compared with reference values obtained from phantoms without scatterers (horizontal black lines).

All three phantom materials demonstrated high mechanical stability across the full range of optical scatterer concentrations used [Figs. 7(a)–7(d)]. Silicone phantoms with ${\mathrm{TiO}}_{2}$ (0.02% to 2% w/v) exhibited a tangent modulus ∼63  kPa, within ±5% of the reference value (63.8 kPa). The tangent modulus of agar phantoms with Intralipid (0.5% to 40% v/v) was at ∼40  kPa, consistent with the reference value of 41.1 kPa. Gelatin phantoms showed similarly stable behavior with both ${\mathrm{TiO}}_{2}$ [Fig. 7(c)] and Intralipid [Fig. 7(d)], maintaining tangent modulus among 6.4 kPa across all scatterer concentrations, matching the reference value of 6.3 kPa. In all cases, measured tangent moduli remained within ±8% of the reference value, with no trends indicating mechanical property variation at higher scatterer concentrations. This mechanical stability across scatterer concentrations, combined with the optical tuning demonstrated in Sec. 4.1.4 (Fig. 6), confirms that mechanical and optical properties can be controlled independently in these phantom materials.

### 4.2 Quantitative Micro-Elastography Measurements

#### 4.2.1 Inclusion phantoms

**Fig. 8:**
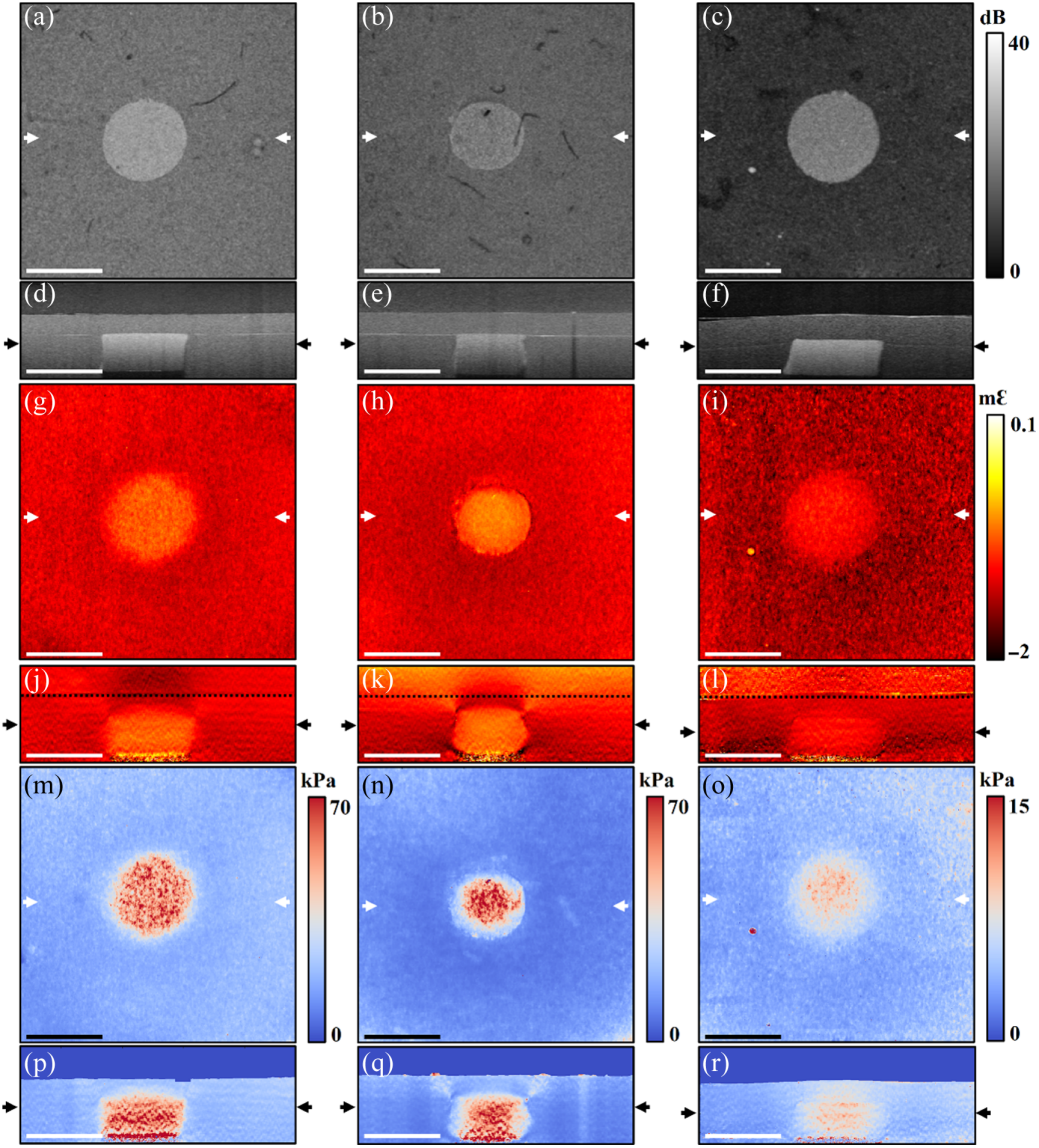
QME measurements of inclusion phantoms fabricated from silicone, agar, and gelatin. The three columns represent the silicone (a), (d), (g), (j), (m), and (p); agar (b), (e), (h), (k), (n), and (q); and gelatin (c), (f), i), (l), (o), and (r) phantoms, each containing a stiff inclusion embedded within a softer bulk matrix. (a)–(c) *En face* OCT intensity images showing the circular inclusion geometry with higher OCT intensity. (d)–(f) Cross-sectional OCT B-scans in the xz plane through the inclusion center. (g)–(i) *En face* axial strain maps at the inclusion plane, displaying reduced strain within the stiffer inclusion compared with the surrounding bulk matrix. (j)–(l) Cross-sectional strain maps in the xz plane, showing the spatial distribution of strain through the inclusion depth, with black dotted lines showing the interface of the compliant layer and phantom. (m)–(o) *En face* elasticity maps. (p)–(r) Cross-sectional elasticity maps. White arrowheads in the *en face* images indicate the location of cross-sectional B-scans shown in panels (d)–(f), (j)–(l), and (p)–(r). Black arrows on cross-sectional images indicate the depth of the corresponding *en face* images. Scale bars represent 1 mm.

Figure 8 presents QME measurements of the inclusion phantoms fabricated from silicone, agar, and gelatin described in Sec. 3.2.2 and Table 2. *En face* OCT intensity images acquired ∼150  μm below the top surface of the inclusions [Figs. 8(a)–8(c)] show well-defined cylindrical inclusions with increased OCT SNR relative to the surrounding bulk, consistent with the higher optical scatterer concentration in the inclusions. Cross-sectional OCT B-scans [Figs. 8(d)–8(f)], positions indicated by arrowheads in the *en face* OCT images, verify the embedded 3D inclusion geometry and continuity of the inclusion through depth.

Axial strain maps demonstrate clear mechanical contrast in all phantoms. *En face* strain maps [Figs. 8(g)–8(i)] show lower strain magnitude within the inclusions relative to the softer bulk matrix, as expected. Strain was measured in $400\;\mu m^{2}\times 400\;\mu m^{2}$ ROIs in the xy plane centered within the inclusion and in the bulk region (positioned 400  μm from the inclusion boundary), both at the same plane shown in the *en face* images. The mean strain values within the inclusion and bulk regions were −0.5±0.1 and −1.0±0.1  mε for silicone, −0.3±0.1 and −1.1±0.1  mε for agar, and −0.8±0.1 and −1.3±0.1  mε for gelatin. Cross-sectional strain maps [Fig. 8(j)–8(l)] confirmed that strain contrast was maintained throughout the full inclusion depth, with bulk-to-inclusion strain ratios of ∼2.1, 3.6, and 1.6 for silicone, agar, and gelatin, respectively. Notably, strain B-scans across all three materials revealed elevated strain in the bulk area directly above each inclusion [Fig. 8(j)–8(l)]. In incompressible materials, such as the three phantom materials studied, the deformation of mechanically bonded adjacent regions is coupled. As the softer bulk region undergoes greater compression than the stiffer inclusion, stress transfer at the boundary results in higher deformation in the bulk directly above the inclusion.^44^^,^^61^

To evaluate QME accuracy in inclusion phantoms, the mean elasticity values from inclusion and bulk regions were compared with reference values from compression testing at near zero strain. This comparison is appropriate because the compliant layer–sample ensemble in QME experiences minimal bulk compression (∼2% to 3% pre-strain), with negligible compressive strain in the inclusion. The silicone inclusion phantom [Fig. 8(m)] showed close agreement with reference values, with inclusion elasticity of 54.6±11.3  kPa compared with the reference of 59.9 kPa (9% error) and bulk elasticity of 16.9±2.0  kPa compared with the reference of 14.0 kPa [20% error, Fig. 8(m)]. The elasticity map revealed smooth transitions from inclusion to bulk, reflecting mechanical coupling at boundaries.^61^

The agar inclusion phantom [Fig. 8(n)] exhibited the largest error. The measured inclusion elasticity (61.7±15.5  kPa) overestimating the reference of 37.9 kPa (2% agar w/v) by 63%, while bulk elasticity (9.1±1.9  kPa) was 28% lower than the reference of 12.7 kPa (1% agar w/v). The elasticity distribution within the inclusion revealed a gradient pattern, with lower elasticity at the periphery compared with the central region [Fig. 8(n)], indicating low mechanical coupling at the inclusion–bulk interface. This peripheral softening and central stiffening pattern is consistent with the strain heterogeneity observed in inclusions with impaired boundary coupling, where restricted stress transmission at the interface produces non-uniform deformation within the inclusion.^288^ Agar exhibits weak interfacial adhesion and does not readily bond to surfaces or other agar structures.^282^ During multi-step casting, as described in Sec. 3.2.2, the pre-cured inclusion is placed on a base layer before the bulk material is poured. Without sufficient cross-linking between the cured inclusion surface and the freshly cast bulk material, mechanical load transfer to the inclusion is impaired. Under compression, this weak interfacial bonding prevents uniform stress transmission, causing the inclusion periphery to appear softer while the center appears stiffer than expected.^61^ The sharper elasticity boundaries and more abrupt transitions observed in the agar inclusion phantom are also consistent with reduced mechanical coupling at the interface of the inclusion.^288^

The gelatin inclusion phantom [Fig. 8(o)] displayed the opposite trend to the agar phantom, with measured inclusion elasticity (8.8±1.0  kPa) being 47% lower than the expected value of 16.6 kPa, while the bulk region elasticity was 4.4±0.6  kPa, 14% lower than the expected value of 5.1 kPa. Despite this underestimation, the phantom maintained high mechanical contrast. The inclusion softening arises from water absorption during fabrication. In the three-step casting protocol, the pre-cured 20% w/v gelatin inclusion is placed in contact with the liquid 10% w/v gelatin bulk solution for ∼30  min before final gelation. This exposure allows water from the bulk solution to absorb into the higher-concentration inclusion, weakening the gelatin network and reducing its stiffness.^284^ Studies have shown that the stiffness of gelatin decreases ∼10-fold as water content increases from 50% to 90% due to hydrogen bond dissociation.^285^ The elasticity map exhibited smooth transitions from inclusion to bulk, similar to the silicone inclusion phantom, indicating mechanical coupling at boundaries.

#### 4.2.2 Surface roughness phantoms

**Fig. 9:**
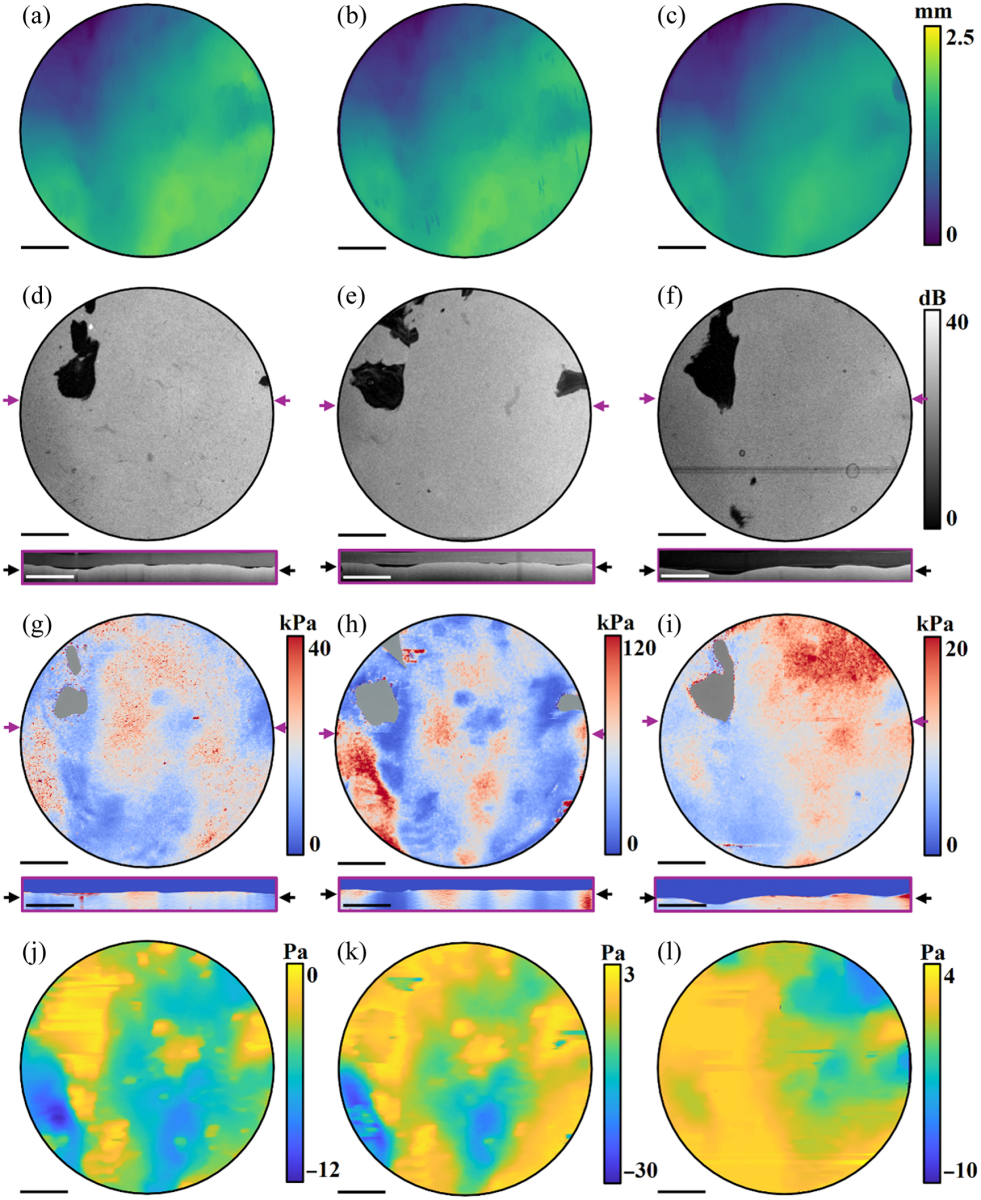
Surface roughness phantoms fabricated from silicone (left column), agar (middle column), and gelatin (right column). (a)–(c) OCT-derived surface height maps showing the replicated breast tissue topography across all three materials. (d)–(f) *En face* OCT intensity images with corresponding cross-sectional B-scans (insets), where dark regions indicate non-contact areas between the rough phantom surface and the imaging window. Purple arrows indicate the location of the corresponding cross-sectional B-scans. (g)–(i) *En face* elasticity maps with corresponding cross-sectional elasticity B-scans (insets). (j)–(l) Bulk stress maps estimated from the compliant layer. Black arrows on cross-sectional images indicate the depth of the corresponding *en face* images. Scale bars represent 2 mm.

Figure 9 presents the surface roughness phantoms, successfully fabricated from all three materials, as described in Sec. 3.3, that replicate the surface topography of excised human breast tissue. To validate the fidelity of surface reproduction, OCT-derived height maps of the phantoms [Figs. 9(a)–9(c)] were compared with the corresponding height map of the excised breast tissue specimen used to generate the mold. The two-dimensional correlation coefficient between the phantom and tissue surface height profiles was 0.93 for silicone, agar, and gelatin, demonstrating that all three materials faithfully replicated the micro-scale surface topography of the breast tissue. These correlation values are consistent with those reported by Sanderson et al.^33^ for surface roughness phantoms fabricated from silicone using the same methodology. This study demonstrates, for the first time, the successful fabrication of surface roughness phantoms from agar and gelatin.

QME measurements of the phantoms fabricated from silicone (left panel), agar (middle panel), and gelatin (right panel) are presented in Figs. 9(d)–9(l). *En face* OCT intensity images [Figs. 9(d)–9(f)] reveal regions of non-contact between the phantom surface and the imaging window, visible as dark areas where the rough topography prevents full contact at the 20% pre-strain applied. This pre-strain was applied from the point of first contact between the highest surface peak (with the compliant layer on top) and the imaging window. Cross-sectional B-scans confirm the non-uniform surface profiles across all three materials, consistent with the breast tissue topography encoded in the 3D-printed molds.

Despite all three phantom materials being mechanically homogeneous, the corresponding elasticity maps [Figs. 9(g)–9(i)] reveal spatially varying elasticity in each of the phantoms. Notably, direct comparison of the elasticity maps [Figs. 9(g)–9(i)] with the surface height maps [Figs. 9(a)–9(c)] and stress maps [Figs. 9(j)–9(l)] reveals that regions of elevated elasticity correspond to surface peaks, where the stress measured in the compliant layer is also elevated. This elevated stress reflects greater local deformation of the compliant layer at surface peaks, which undergo greater compression under axial loading than surrounding valleys. This trend is observed across all three materials and arises from the non-uniform stress imposed by the rough surface geometry rather than true mechanical heterogeneity within the phantom, as described by Sanderson et al.^33^

This effect introduces apparent mechanical contrast in a homogeneous phantom, demonstrating that surface roughness alone can produce false elasticity contrast in compression OCE, an important consideration when interpreting elastograms of tissues with complex surface topography such as excised breast specimens. The gray regions visible in the *en face* elasticity maps correspond to areas of negative elasticity, which arise from positive strain measured in the compliant layer or surface roughness phantom at low-height regions (valleys). At these locations, the compliant layer or phantom undergoes tensile rather than compressive deformation due to the surface geometry. An additional factor influencing the elasticity measured in surface roughness phantoms is the distribution of lubricants across the surface. Surface peaks cause lubricants to pool in local valleys, leaving only a thin film at the peaks. The resulting spatially varying friction at the layer–sample interface modifies the measured strain and stress estimated in QME, contributing to both overestimation and underestimation of elasticity across the phantom surface. The combination of these effects, variable pre-strain, elevated stress at surface peaks, and variable surface friction, results in the heterogeneous elasticity patterns observed in Figs. 9(g)–9(i), despite the mechanical uniformity of the phantoms.

To investigate whether increasing pre-strain could improve contact between the rough phantom surface and the imaging window, we applied pre-strains of 30% and 40% to the surface roughness phantoms made from silicone using a stiffness-matched compliant layer. Despite the increased pre-strain, the contact area between the phantom surface and the compliant layer did not increase appreciably, as evidenced by the persistence of dark non-contact regions in the *en face* OCT intensity images [Figs. S1(a)–S1(c) in the Supplementary Material].

To address this, a softer compliant layer was substituted while maintaining a pre-strain of 30%. Under this condition, a larger proportion of the phantom surface achieved contact with the imaging window [Figs. S1(d)–S1(f) in the Supplementary Material], reducing the extent of non-contact regions visible in the OCT image. This improvement is attributed to the softer layer allowing more uniform load redistribution across the rough surface, promoting greater contact than with the stiffer layer. These findings highlight that compliant layer stiffness not only affects the accuracy of stress estimation in QME (as described in Sec. 4.2.1) but also influences the effective contact area achievable on surface roughness phantoms, an important practical consideration when imaging tissue.

## 5 Discussion

In this study, silicone, agar, and gelatin, the three most widely used phantom materials in OCE were characterized. We measured the properties most relevant to phantom design, namely, elasticity, viscoelasticity, optical attenuation, temporal stability, and suitability for use as structured phantoms. The results showed that no single material is optimal for all applications. Instead, each material offers a different balance among these properties, highlighting the need for application-specific phantom designs and improved standardization in OCE phantom development.

Across the three materials, the achievable range of tangent modulus differed substantially. Silicone covered a broad range of 7.2 to 175.9 kPa and provided the best repeatability under repeated loading, the greatest temporal stability, and the most reliable fabrication performance, consistent with prior studies.^289^ Stress relaxation testing further showed that silicone was predominantly elastic. Together, these properties make silicone particularly well suited for durable, reproducible phantoms, especially where mechanical stability and repeatable measurements are required.^26^^,^^290^

Agar covered the broadest range from 9.7 to 229.1 kPa, providing flexibility for fabricating stiffer phantoms. Stress relaxation testing also showed that, among the materials tested, agar exhibited the closest agreement with commonly reported soft tissue viscoelastic behavior, with rapid relaxation and substantial viscous dissipation.^291^^,^^292^ However, agar phantoms did not fully recover after first loading, likely due to plastic deformation and moisture loss under compression, which reduced the repeatability of mechanical testing.

Gelatin produced the softest phantoms in this study, with a tangent modulus range of 3.5 to 29.4 kPa, making it particularly advantageous for mimicking very soft tissues. The relatively low stiffness of the gelatin phantoms compared with previously reported values is likely explained by the testing protocol used here, as gelatin phantoms were equilibrated at room temperature for 1 h before testing. This equilibration period allowed the samples to reach a stable room temperature mechanical state but also resulted in lower measured stiffness than would be expected if testing were performed immediately after refrigeration. Stress relaxation testing showed that gelatin had the slowest viscoelastic response. This may be useful in applications where slow relaxation or pronounced viscoelasticity is desired. However, the narrower stiffness range may limit its use. Gelatin was also more sensitive to storage and testing conditions, requiring room temperature equilibration and characterization within 1 to 2 days of fabrication.

Overall, agar and gelatin offer a key advantage over silicone in applications where pronounced viscoelastic behavior is required, while silicone offers the strongest performance where durability, repeatability, temporal stability, and fabrication reliability are the main priorities.

Optical tuning through the addition of scattering agents was achievable in all three materials, with comparable maximum attenuation coefficients (∼10 to $13\;{\mathrm{mm}}^{-1}$) reached across silicone, agar, and gelatin, overlapping with attenuation coefficients reported for soft biological tissues.^31^ More broadly, it addresses a common oversight in previous OCE studies, where scatterer concentrations are often reported without characterization of the resulting attenuation coefficient. Measuring attenuation improves reproducibility and makes phantom fabrication more transferable across studies and systems.

The property ranges achieved across the three materials overlap with those of soft biological tissues. The measured tangent moduli (3.5 to 229.1 kPa) fall within the 0.1 to 1000 kPa range reported for soft tissues.^28^ For example, the Young’s modulus of adipose tissue has been measured in the range of ∼1 to 3 kPa,^293^ fibrous connective tissues have been reported in the range ∼10 to 50 kPa,^294^ and many tumors are ∼50 to 300 kPa.^295^ Individual phantom formulations reported in this work can therefore be selected to replicate the stiffness of specific tissues. The achievable OCT attenuation coefficients (0.2 to $13.1\;{\mathrm{mm}}^{-1}$) also overlap with the 1.6 to $16.5\;{\mathrm{mm}}^{-1}$ range reported for soft tissues.^31^ Attenuation in weakly scattering tissues such as adipose regions is generally lower ($<2\;{\mathrm{mm}}^{-1}$)^296^ and higher in skin, muscle, and fibrous connective tissues with measurements typically ranging from 5 to $15\;{\mathrm{mm}}^{-1}$.^31^ Soft tissues also show pronounced viscoelastic behavior, relaxing over timescales of tens to hundreds of seconds.^291^^,^^292^ For example, it has been reported that adipose exhibited a fast relaxation time constants ranging between 120 and 240 s.^293^ In addition, a relaxation time constant of 66 s has been reported in pancreatic tumors.^297^ Agar and gelatin reproduced this response, whereas silicone was predominantly elastic and better suited to applications needing a stable elastic response. Overall, the three materials can replicate the mechanical and optical property ranges of tissues relevant to OCE.

Regarding structured phantoms, we demonstrated that all three materials could be used to fabricate inclusion phantoms. Silicone was the most robust, as thin agar and gelatin inclusions are prone to tearing, dehydration, and shrinkage during fabrication and handling. Material-specific considerations highlight the importance of the fabrication protocol for achieving reliable mechanical properties in heterogeneous phantoms. For agar phantoms, surface treatments or modified curing strategies that promote interfacial cross-linking between the pre-cured inclusion and bulk material may improve mechanical integration and, therefore, the robustness and accuracy of OCE measurements. For gelatin, minimizing the time between casting steps and controlling hydration states of both components during assembly could reduce water-mediated softening. Despite these considerations, QME was able to successfully resolve the intended mechanical contrast and geometric features across all three materials, demonstrating its utility for phantom characterization while revealing fabrication-dependent mechanical artifacts that must be considered when designing structured phantoms for OCE validation.

In QME, surface roughness phantoms also highlighted the effect of compliant layer stiffness on contact quality. In this study, the effects of pre-strain and compliant layer stiffness were investigated on silicone phantoms. Agar surface roughness phantoms were not subjected to higher pre-strains, as these gels were found to fracture under compressive strains above 20%. Although gelatin phantoms can withstand higher pre-strains without fracture, the soft and thin compliant layers are prone to tearing during handling. In future work, through careful design of both phantoms and layers, it may be feasible to extend this analysis to agar and gelatin. These findings are relevant to OCE validation, since OCE imaging performance depends not only on the intrinsic mechanical properties of the phantom but also on its structural properties, such as surface roughness, under realistic loading and imaging conditions.

The structured phantoms were imaged using a single compression OCE implementation, but the observed effects are not all system-specific. The apparent elasticity contrast from surface roughness is specific to compression OCE. However, a related geometric effect has been reported in wave-based OCE, where sample curvature alters the measured wave velocity.^160^ Boundary effects, where the degree of bonding at an inclusion changes the local strain distribution, are documented in both quasi-static ultrasound elastography^288^ and compression OCE.^61^ The deviations in the agar and gelatin inclusion phantoms arise from weak interfacial bonding and hydration-related softening. These are material effects rather than artifacts of the specific QME implementation, although their appearance and magnitude may depend on the loading and image formation method. The phantom elasticity was independently validated against uniaxial compression testing, providing a system-independent reference for the QME measurements. Applying these phantoms across compression, transient, and harmonic OCE would establish which effects are common to all methods and which are specific to a particular OCE system.

Importantly, this study established, for the first time, a framework for fabricating and evaluating structured phantoms across silicone, agar, and gelatin. In particular, we extended structured OCE phantom design beyond the predominantly silicone-based approaches reported previously. Although the present work focused on uniaxial compression and stress relaxation testing, future studies could incorporate complementary characterization approaches, such as rheometry and indentation, to provide a broader assessment of phantom behavior. Contact optimization on rough surfaces was investigated systematically in silicone only; extending this to agar and gelatin remains an area for future work, requiring solutions to challenges such as fracture at higher pre-strains and handling of the compliant layers made with hydrogels. Assessment of structured phantoms using other OCE techniques would also provide further insight into phantom performance under different loading and imaging conditions. In summary, the results presented in this paper provide a foundation for expanding phantom design to additional materials and application-specific geometries while supporting the development of standardized fabrication and characterization protocols for OCE validation.

## 6 Conclusion

This study presented a systematic characterization of silicone, agar, and gelatin, covering mechanical, optical, and structural properties. Silicone and agar demonstrated broad tunability in elasticity, while gelatin covered a narrower elasticity range. All three materials exhibited distinct, tunable viscoelastic signatures. Optical attenuation was tunable independently of mechanical properties, encompassing most soft tissues relevant to OCE. Surface roughness phantoms were successfully fabricated from all three materials and accurately reproduced the surface topography of human breast tissue, with QME measurements further demonstrating that surface topography alone can produce apparent mechanical contrast in compression OCE elastograms. Overall, silicone is best suited to durable, reproducible phantoms requiring long-term stability and predominantly elastic behavior; agar to viscoelastic hydrogel phantoms spanning a broad elasticity range, stored in water to maintain stability; and gelatin to soft or strongly viscoelastic phantoms, best characterized soon after fabrication. Together, these results provide a framework to guide phantom material selection that we believe can advance standardization in OCE phantom development.

## Supplementary Material

10.1117/1.JBO.31.8.086004.s01

## Data Availability

The data underlying the results presented in this paper, together with the custom MATLAB analysis scripts, are not publicly available at this time but may be obtained from the corresponding author upon reasonable request.
