# Supplementary material for: Phantoms for use in optical coherence elastography
Source: J Biomed Opt. 2026 Aug 8;31(8):086004. doi: 10.1117/1.JBO.31.8.086004 (PMC13452405; doi:10.1117/1.JBO.31.8.086004)
Supplement: Supplementary file 1 [file JBO_031_086004_SD001.pdf]

Supplementary material for “Phantoms for use in optical coherence elastography”

This supplementary material provides additional data supporting the findings reported in the main manuscript.

Table S1. OCE phantom literature review (223 studies, 1998–2025). OCE: optical coherence elastography. Reference numbers correspond to those in the main manuscript.

Year	Title	Phantom Material	OCE Type	Phantom Structure	Optical Scattering	Ref
2025	Schumacher, *et al.*	Agar	Transient	Homogeneous	Intralipid	108
2025	Singh, *et al.*	Agar	Transient	Homogeneous	–	109
2025	Chavez, *et al.*	Silicone & Agar	Compression	Inclusion	TiO_2	34
2025	Lopez, *et al.*	Agarose	Transient & Harmonic	Homogeneous	–	110
2025	Zhou, *et al.*	Agar		Homogeneous	–	111
2025	Navaeipour, *et al.*	Silicone	Compression	Tissue-mimicking	TiO_2	32
2025	Duvvu, *et al.*	Silicone	Transient	Homogeneous	–	35
2025	Yang, *et al.*	Gelatin & Silicone	Other	–	–	36
2025	Song, *et al.*	Agar	Transient	Homogeneous	–	112
2025	Chen, *et al.*	Agarose	Transient	Homogeneous & Layered	–	113
2025	Jones, *et al.*	Silicone	Compression	Homogeneous & Inclusion	TiO_2	37
2024	Singh, *et al.*	Gelatin	Harmonic	Homogeneous & Layered	Intralipid	181
2024	Navaeipour, *et al.*	Silicone	Compression	Homogeneous	TiO_2	38
2024	Sanderson, *et al.*	Silicone	Compression	homogenous & tissue-mimicking	TiO_2	33
2024	Foo, *et al.*	Silicone	Compression	Inclusion	–	39
2024	Metzner, *et al.*	Silicone	Compression	Homogeneous & inclusion	TiO_2	40
2024	Han, *et al.*	Agar	Transient	Layered	–	114
2024	Shi, *et al.*	Agar	Transient	Homogeneous	–	115
2024	Zhang, *et al.*	Agar	Transient	Layered	Intralipid	116
2024	Zhang, *et al.*	Agar	Harmonic	Layered	Intralipid	117

2024	Neidhardt, *et al.*	Gelatin	Harmonic	Homogeneous & Inclusion	TiO_2	182
2024	Zhang, *et al.*	Agar	Transient	Homogeneous	–	118
2024	Feng, *et al.*	Silicone	Transient	Homogeneous	White pigment	41
2024	Burhan, *et al.*	PVA*	Transient	Homogeneous	–	228
2024	Asemani, *et al.*	Gelatin	Harmonic	Inclusion	Intralipid	183
2024	Liu, *et al.*	CIRS	Transient	Homogeneous	Silicon carbide	244
2024	Liu, *et al.*	–	Transient	Homogeneous	–	244
2024	Xu, *et al.*	Gelatin & Agar	Transient	Homogeneous	Intralipid	184
2024	Ma, *et al.*	Agar	Transient	Homogeneous	Intralipid	119
2024	Song, *et al.*	Agar	Transient	Homogeneous	–	120
2024	Wang, *et al.*	Agar	Transient	Homogeneous & Layered	–	121
2024	Wang, *et al.*	Agar	Transient	–	–	122
2024	Xu, *et al.*	Hydrogel	Harmonic	Homogeneous	TiO_2	245
2024	Liu, *et al.*	Agar	Harmonic	Homogeneous	Intralipid	123
2024	Zhang, *et al.*	Silicone	Compression	Vessel-like	Alumina powder	42
2024	Parmar, *et al.*	Agar	Transient	Homogeneous	–	124
2023	Zhu, *et al.*	Agar	Transient	Homogeneous	–	125
2023	Zhu, *et al.*	Agar	Transient	Homogeneous	Intralipid	126
2023	Batista, *et al.*	Agarose	Transient	Homogeneous	–	127
2023	Zhang, *et al.*	Agar	Transient	Homogeneous	Intralipid	128
2023	Latus, *et al.*	Gelatin	Transient	Homogeneous & Layered	TiO_2	185
2023	Liu, *et al.*	Silica gel	Transient	Homogeneous	TiO_2	239
2023	Li, *et al.*	Gelatin	Transient	Homogeneous	Silicon carbide	186
2023	Fang, *et al.*	Silicone	Compression	Inclusion	TiO_2	43
2023	Metzner, *et al.*	Silicone	Compression	Homogeneous & Inclusion	TiO_2	44
2023	Li, *et al.*	Silicone	Compression	Homogeneous & circular cut-out	TiO_2	45
2023	Hatami, *et al.*	PAA**	Transient	Layered	–	236
2023	Mekonnen, *et al.*	Gelatin	Transient	Layered	–	187
2023	Wang, *et al.*	Agar	Transient	Homogeneous	Intralipid	129

2023	Lin, *et al.*	Silica gel	Compression	Homogeneous & Layered	TiO_2	240
2023	Neidhardt, *et al.*	Silicone	Transient	Homogeneous	TiO_2	46
2022	Regnault, *et al.*	–	Transient	Layered	TiO_2	246
2022	Li, *et al.*	Agar	Transient	Homogeneous	–	130
2022	Li, *et al.*	Silicone	Compression	Homogeneous & Inclusion	TiO_2	47
2022	Krainz, *et al.*	Silicone	Compression	Inclusion	TiO_2	48
2022	Wang, *et al.*	PVA	Harmonic	Inclusion	–	229
2022	Bu, *et al.*	Silicone	Compression	Homogeneous	Al2O3	49
2022	Wang, *et al.*	–	Compression	Layered	–	247
2022	Yang, *et al.*	Agar	Harmonic	Layered	TiO_2	131
2022	Lin, *et al.*	Silica gel	Compression	Homogeneous	TiO_2	241
2022	Gao, *et al.*	Gelatin	Compression	Homogeneous	–	188
2022	Han, *et al.*	Agar	Transient	Homogeneous	Intralipid	132
2021	Parmar, *et al.*	Agar	Transient	Homogeneous	Milk	133
2021	Lu, *et al.*	Gelatin	Transient	Homogeneous & Layered	Silicon carbide	189
2021	Kirby, *et al.*	–	Transient	Homogeneous	–	248
2021	Ormachea, *et al.*	CIRS	Harmonic	Layered	Intralipid	249
2021	Hepburn, *et al.*	Silicone	Compression	Homogeneous & Inclusion	TiO_2	50
2021	Li, *et al.*	Silicone	Compression	Homogeneous & Inclusion	TiO_2	20
2021	Fang, *et al.*	Silicone	Compression	Inclusion	TiO_2	51
2021	Sanderson, *et al.*	Silicone	Compression	Inclusion	TiO_2	52
2021	Singh, *et al.*	Gelatin	Transient	Homogeneous	TiO_2	190
2021	Rippy, *et al.*	Gelatin	Transient	Homogeneous	Black paint	191
2021	Chen, *et al.*	Silica gel	Harmonic	Layered	TiO_2	242
2021	Liu, *et al.*	Gelatin	Transient & Harmonic	Homogeneous & Inclusion	TiO_2	192
2021	Zhou, *et al.*	Agar	Harmonic	Layered	–	134
2020	Pitre, *et al.*	PVA	Transient	Homogeneous	TiO_2	230
2020	Huang, *et al.*	Silicone	Other	Homogeneous & Layered	TiO_2	53
2020	Neidhardt, *et al.*	Gelatin	Harmonic	Homogeneous	–	193
2020	Das, *et al.*	Graphite	Transient	Homogeneous	–	250
2020	Zvietcovich, *et al.*	Agar	Harmonic	Homogeneous & Layered	–	135

2020	Alexandrovskaya, *et al.*	Silicone	Compression	Homogeneous	–	54
2020	Boerner, *et al.*	PAA	Transient	Layered	Intralipid	237
2020	Zvietcovich, *et al.*	Agar	Transient	Homogeneous	–	136
2020	Sovetsky, *et al.*	Silicone	Compression	Homogeneous	–	55
2020	Liu, *et al.*	Gelatin	Transient	Layered	TiO_2	194
2020	Zhang, *et al.*	Agar	Harmonic	Homogeneous	TiO_2	137
2020	Yao, *et al.*	Agar	Compression	Homogeneous	Intralipid	138
2020	Kang, *et al.*	Gelatin	Harmonic	Homogeneous	Silicon carbide	195
2020	Sanderson, *et al.*	Silicone	Compression	Inclusion	TiO_2	56
2020	Fang, *et al.*	Silicone	Compression	Homogeneous & Inclusion	TiO_2	57
2020	Hepburn, *et al.*	Silicone	Compression	Homogeneous & Inclusion	TiO_2	58
2020	Lan, *et al.*	Agar	Transient	Homogeneous	–	139
2020	Ambekar, *et al.*	Gelatin	Transient	Homogeneous	–	196
2020	Lan, *et al.*	Agar	Transient	Homogeneous	Graphite powder	180
2020	Li, *et al.*	–	Compression	Homogeneous	–	251
2020	Qian, *et al.*	Silicone	Transient	Homogeneous	–	59
2020	Zhang, *et al.*	Gelatin & Agar	Harmonic	Homogeneous & Layered	TiO_2	197
2019	Huang, *et al.*	Silicone	Harmonic	Homogeneous & Inclusion	TiO_2	60
2019	Pitre, *et al.*	PVA	Transient	Homogeneous & Layered	–	231
2019	Bu, *et al.*	–	Compression	tube	–	252
2019	Zvietcovich, *et al.*	Gelatin	Harmonic	Homogeneous & Layered	Intralipid	198
2019	Hepburn, *et al.*	Silicone	Compression	Inclusion	TiO_2	61
2019	Zaitsev, *et al.*	Silicone	Compression	Homogeneous	–	62
2019	Zhang, *et al.*	Agar & Gelatin	Transient	Homogeneous	–	140
2019	Meng, *et al.*	Silica gel	Compression	Homogeneous	TiO_2	243
2019	Jin, *et al.*	Agar	Transient	Homogeneous	Intralipid	141
2019	Liou, *et al.*	Agarose	Harmonic	–	Milk	142
2019	Zvietcovich, *et al.*	Gelatin	Harmonic	Layered	–	199
2019	Zvietcovich, *et al.*	CIRS	Harmonic	Homogeneous	–	253

2019	Kirby, *et al.*	PVA	Transient	Layered	TiO_2	19
2019	Liu, *et al.*	PAA	Transient	Layered	Intralipid	238
2019	Fang, *et al.*	Silicone	Compression	Homogeneous & Inclusion	TiO_2	63
2019	Dong, *et al.*	Silicone	Compression	Inclusion	TiO_2	64
2019	Krajancich, *et al.*	Silicone	Compression	Inclusion	TiO_2	65
2019	Sanderson, *et al.*	Silicone	Compression	Homogeneous & Layered & Inclusion	TiO_2	66
2018	Wu, *et al.*	Agar	Transient	Homogeneous	–	143
2018	Wijesinghe, *et al.*	Silicone	Compression	Inclusion & Cavity	TiO_2	67
2018	Chen, *et al.*	Gelatin	Transient	Layered	Graphite powder	200
2018	Zhu, *et al.*	Agar	Transient	Homogeneous & Layered	Intralipid	144
2018	Chen, *et al.*	Agarose	Other	Homogeneous & Layered	Intralipid	145
2018	Qu, *et al.*	Silicone	Transient	Homogeneous	–	68
2018	Shih, *et al.*	Gelatin & Agar	Transient & Harmonic	Layered	–	201
2018	Qu, *et al.*	Silicone	Transient	Homogeneous	–	69
2018	Zhu, *et al.*	Agar	Transient	Homogeneous & Layered	Intralipid	146
2018	Zhou, *et al.*	Liquid paraffin cream in agar	Transient	Homogeneous	–	147
2018	Sovetsky, *et al.*	Silicone	Compression	Homogeneous	–	70
2018	Zhou, *et al.*	Agar	Transient	Homogeneous	TiO_2	148
2018	Liu, *et al.*	Agar	Transient	Homogeneous	–	149
2018	Karpiouk, *et al.*	Gelatin	Transient	Homogeneous	Graphite powder	202
2017	Zvietcovich, *et al.*	CIRS	Transient	Homogeneous	–	254
2017	Qu, *et al.*	Gelatin	Transient	Layered	–	203
2017	Zvietcovich, *et al.*	Gelatin	Transient	Layered	Intralipid	204
2017	Chae, *et al.*	Silicone	Compression	Homogeneous	TiO_2	71
2017	Es'haghian, *et al.*	Silicone	Compression	Inclusion	TiO_2	72
2017	Fang, *et al.*	Silicone	Compression	Inclusion	TiO_2	73
2017	Lan, *et al.*	Agar	Transient	Homogeneous	–	150

2017	Fang, *et al.*	Silicone	Compression	Inclusion	TiO_2	74
2017	Singh, *et al.*	Agar	Transient	Homogeneous & Inclusion	–	151
2017	Liu, *et al.*	Agar	Transient	Homogeneous & Layered	–	152
2016	Allen, *et al.*	Silicone	Compression	Inclusion	TiO_2	75
2016	Curatolo, *et al.*	Silicone	Compression	Inclusion	TiO_2	76
2016	Zhu, *et al.*	Agar	Transient	Layered	Intralipid	153
2016	Larin, *et al.*	Polyacrilamide hydrogel	Compression	Homogeneous	–	23
2016	Wu, *et al.*	Agar	Transient	Homogeneous & Layered	Milk	154
2016	Han, *et al.*	Gelatin	Transient	Homogeneous	Milk	205
2016	Zaitsev, *et al.*	–	Compression	Layered	–	255
2016	Nguyen, *et al.*	Agarose	Other	Layered	TiO_2	155
2016	Qiu, *et al.*	Silicone	Compression	Homogeneous	TiO_2	77
2016	Qu, *et al.*	Agarose	Harmonic	Inclusion	–	156
2016	Qiu, *et al.*	Silicone	Compression	Homogeneous	–	78
2016	Liu, *et al.*	Agar	Transient	Layered	–	157
2016	Hsieh, *et al.*	Gelatin	Transient	Layered & Inclusion	TiO_2	206
2016	Dong, *et al.*	Silicone	Compression	Inclusion	—	84
2016	Meemon, *et al.*	Gelatin	Harmonic	Layered	Intralipid	207
2016	Parasaram, *et al.*	Agar	Transient	Homogeneous & Layered	Intralipid	158
2016	Huang, *et al.*	Agarose	Other	Layered	–	159
2016	Zaitsev, *et al.*	Silicone	Compression	Layered	–	79
2015	Es'haghian, *et al.*	Silicone	Compression	Layered with Inclusion	TiO_2	80
2015	Kirk, *et al.*	Silicone	Compression	Inclusion	TiO_2	81
2015	Nguyen, *et al.*	Gelatin	Transient	Homogeneous & Layered	Latex microspheres	208
2015	Ahmad, *et al.*	Silicone	Other	Layered	TiO_2	82
2015	Han, *et al.*	Agar	Transient	Homogeneous	–	160
2015	Singh, *et al.*	Agar	Transient	–	–	161
2015	Kennedy, *et al.*	Silicone	Compression	Homogeneous & Layered & Inclusion	TiO_2	83

2015	Han, *et al.*	Agar	Transient	Homogeneous	–	162
2015	Han, *et al.*	Agar	Transient	Homogeneous	–	163
2015	Singh, *et al.*	Agar	Transient	Homogeneous	Ink	164
2015	Aglyamov, *et al.*	Gelatin	Transient	Homogeneous & Layered	–	209
2015	Song, *et al.*	Agarose	Transient	Homogeneous	TiO_2	165
2015	Wijesinghe *et al.*	Silicone	Compression	Homogeneous & Layered	TiO_2	85
2015	Ma, *et al.*	Agar	Harmonic	Layered & Inclusion	–	166
2014	Nguyen, *et al.*	Agar	Transient	Homogeneous & Inclusion	Latex spheres	167
2014	Ahmad, *et al.*	Gelatin & Agar	Other	Homogeneous	TiO_2	210
2014	Kennedy, *et al.*	Silicone	Compression	Homogeneous & Inclusion	TiO_2	86
2014	Zaitsev, *et al.*	Silicone	Compression	Homogeneous	–	87
2014	Kennedy, *et al.*	Silicone	Compression	Inclusion	TiO_2	88
2014	Chin, *et al.*	Silicone	Compression	Inclusion	TiO_2	89
2014	Wang, *et al.*	Agar	Transient	Homogeneous	–	168
2014	Qi, *et al.*	Silicone	Transient	Homogeneous	–	90
2014	Li, *et al.*	Agar	Other	Homogeneous	ink	169
2014	Razani, *et al.*	Gelatin	Transient	Layered	TiO_2	211
2014	Li, *et al.*	Gelatin	Transient	–	–	212
2014	Kennedy, *et al.*	Silicone	Compression	Inclusion	TiO_2	91
2014	Li, *et al.*	Agar & Gelatin	Transient	Homogeneous	–	170
2013	Kennedy, *et al.*	Silicone	Compression	Homogeneous & Layered & Inclusion	TiO_2	21
2013	Li, *et al.*	Gelatin	Transient	Homogeneous	–	213
2013	Nahas, *et al.*	PVA & Agarose	Other	Homogeneous	silver paint, zinc oxide	232
2013	Wang, *et al.*	Gelatin	Transient	Homogeneous	–	214
2013	Wang, *et al.*	Gelatin	Transient	Homogeneous & Layered	–	215
2013	Zaitsev, *et al.*	Silicone	Compression	Layered	–	92
2013	Sun, *et al.*	Silicone	Compression	Layered & Inclusion	TiO_2	93

2013	Nahas, *et al.*	Silicone	Other	Layered	Zinc oxide	94
2013	Song, *et al.*	Agar	Transient	Homogeneous & Layered	Milk	171
2013	Song, *et al.*	Agar	Transient	Homogeneous & Inclusion	Milk	172
2013	Guan, *et al.*	Agar	Transient	Layered & Inclusion	Milk	173
2013	Qi, *et al.*	Silicone & Agar	Transient	–	–	95
2012	Kennedy, *et al.*	Silicone	Compression	Inclusion	TiO_2	96
2012	Li, *et al.*	Agar	Transient	Layered & Inclusion	Intralipid	174
2012	Li, *et al.*	Agar	Transient	Layered & Inclusion	Milk	175
2012	Razani, *et al.*	Gelatin	Transient	Homogeneous	TiO_2	216
2012	Qi, *et al.*	Agarose	Transient	Layered	–	176
2012	Manapuram, *et al.*	Gelatin	Transient	–	–	217
2012	Kennedy, *et al.*	Silicone	Compression	Homogeneous & Inclusion	TiO_2	97
2012	Kennedy, *et al.*	Silicone	Compression	Homogeneous & Layered & Inclusion	TiO_2	98
2012	Wang, *et al.*	Gelatin	Transient	Homogeneous	–	218
2012	Li, *et al.*	Agar	Transient	Homogeneous & Layered	Milk	177
2011	Srivastava, *et al.*	Gelatin & Agarose	Other	Homogeneous	Charcoal particles	219
2011	Ford, *et al.*	–	Compression	Homogeneous	–	256
2010	Kennedy, *et al.*	Silicone	Compression	Layered	TiO_2	99
2010	Adie, *et al.*	Silicone	Harmonic	Homogeneous & Inclusion	Polystyrene	100
2010	Liang, *et al.*	Silicone	Harmonic	Layered	Polystyrene	101
2010	Liang, *et al.*	Silicone	Harmonic	Layered	TiO_2	102
2009	Kennedy, *et al.*	Silicone	Harmonic	Layered	TiO_2	103
2009	Adie, *et al.*	Silicone	Harmonic	3-layered	–	104
2009	Liang, *et al.*	Gelatin	Harmonic	Inclusion	–	220
2009	Crecea, *et al.*	Silicone	Other	Homogeneous	TiO_2	105
2009	Huang, *et al.*	Silicone	Other	Homogeneous	–	106
2008	Liang, *et al.*	Silicone	Harmonic	Homogeneous	TiO_2	107

2007	Wang, *et al.*	PVA	Compression	Layered	–	233
2006	Wang, *et al.*	PVA	Compression	Layered	–	234
2006	Kirkpatrick, *et al.*	PVA	Compression	Layered	–	235
2006	Rogowska, *et al.*	Agar & Gelatin	Other	Homogeneous	–	178
2004	Rogowska, *et al.*	Agarose & Gelatin	Other	Homogeneous	Charcoal	179
1998	Schmitt, *et al.*	Gelatin	Compression	Inclusion	Latex spheres	221

*PVA: Polyvinyl alcohol
**PAA: Polyacrylamide

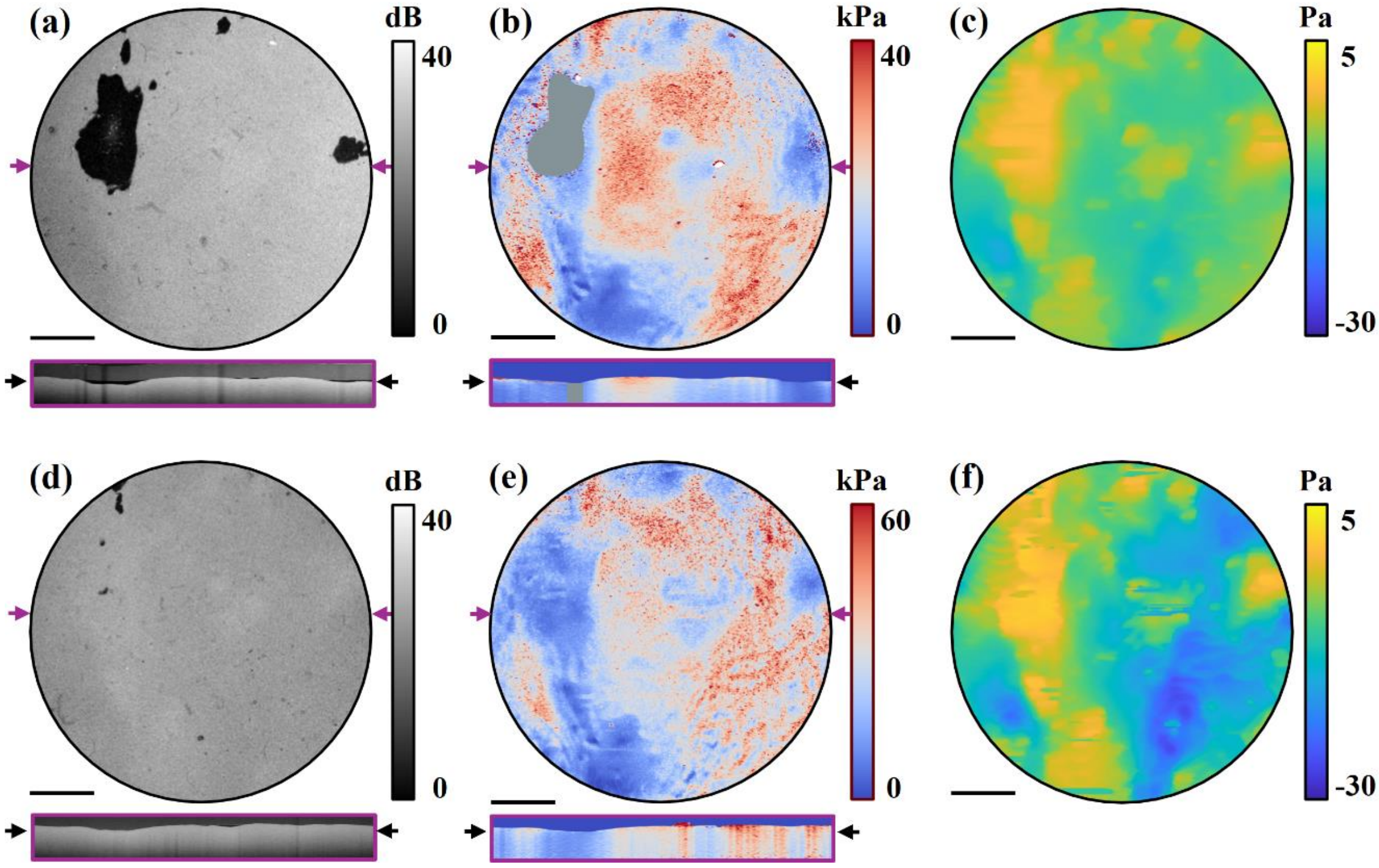

Fig. S1 Effect of compliant layer stiffness and pre-strain on contact area in silicone surface roughness-mimicking phantoms. Top row (a–c): stiffness-matched compliant layer with 30% pre-strain. Bottom row (d–f): softer compliant layer with 30% pre-strain. (a, d) *En face* OCT intensity images showing the extent of contact between the phantom surface and the compliant layer, with dark regions indicating non-contact areas. (b, e) *En face* elasticity maps. Gray regions in the elasticity maps indicate areas of negative elasticity which and have been masked out. (c, f) Bulk stress maps estimated from the compliant layer. Purple arrowheads indicate the location of cross-sectional B-scans. With a stiffness-matched layer (top row), increasing pre-strain to 30% does not appreciably increase the contact area, as evidenced by the persistence of dark non-contact regions in (a). Substituting a softer compliant layer at the same pre-strain (bottom row) results in a larger proportion of the phantom surface achieving contact with the compliant layer (d), accompanied by increased and more spatially distributed stress in the compliant layer (f). Black arrows on cross-sectional images indicate the depth of the corresponding *en face* images. Scale bars represent 2 mm.
